# Embryonic stem cell-derived mesenchymal stem cells promote colon epithelial integrity and regeneration by elevating circulating IGF-1 in colitis mice

**DOI:** 10.7150/thno.47683

**Published:** 2020-10-30

**Authors:** Jun Xu, Xiaofang Wang, Jiaye Chen, Shengbo Chen, Zhijun Li, Hongbin Liu, Yang Bai, Fachao Zhi

**Affiliations:** 1 Guangdong Provincial Key Laboratory of Gastroenterology, Institute of Gastroenterology of Guangdong Province, Department of Gastroenterology, Nanfang Hospital, Southern Medical University, Guangzhou 510515, China.; 2 ImStem Biotechnology, Inc., 400 Farmington Avenue R1808, Farmington, CT 06030, USA.; 3 ZhuHai Hengqin ImStem Biotechnology Co., Ltd, Hengqin New District Huandao Donglu 1889 Building 3, Zhuhai 519000, China.

**Keywords:** mesenchymal stem cell, inflammatory bowel disease, insulin-like growth factor-1, epithelium regeneration, mucosa integrity

## Abstract

**Rationale:** Mesenchymal stem cells (MSCs) show promising therapeutic potential in treating inflammatory bowel disease (IBD) due to their immunomodulatory and trophic functions. However, their efficacy is influenced by tissue origin, donator condition, isolation, and expansion methods. Here, we generated phenotypically uniform MSCs from human embryonic stem cells (T-MSCs) and explored the molecular mechanisms involved in promoting mucosal integrity and regeneration in colitis mice.

**Methods:** T-MSCs were injected intravenously into mice with dextran sulfate sodium (DSS)-induced colitis, and the *in vivo* distribution and therapeutic efficacy were evaluated. We performed serum cytokine antibody microarrays to screen potentially effective proteins and examined the therapeutic effect of insulin-like growth factor-1 (IGF-1). Colon epithelial regeneration potential was evaluated, and RNA sequencing was employed to determine the underlying molecular mechanisms. Finally, *in vitro* IGF-1 stimulation was performed to assess its effect on cell functions and organoid growth.

**Results:** Intravenous administration of T-MSCs alleviated colitis in both acute and chronic DSS mouse models. Labeled T-MSCs were mainly distributed in the lungs, liver, and spleen after systemic infusion. The antibody array analysis of serum cytokines indicated that the IGF-1 level was increased in the treatment group, and serum ELISA further confirmed its elevation in the regeneration stage. Intraperitoneal injection of IGF-1 receptor inhibitors abrogated the anti-inflammatory activity of T-MSCs. The colonic epithelium of the treatment group showed greater regenerative potency than the controls and the IGF1R-PI3K-AKT pathway was up-regulated. RNA sequencing showed that T-MSC treatment contributed to colonic cell integrity and promoted xenobiotic metabolism. *In vitro* IGF-1 stimulation promoted the growth and proliferation of colon cells and organoids.

**Conclusions:** Intravenous infusion of T-MSCs alleviated colitis in mice by elevating the circulating IGF-1 level. Increased IGF-1 maintained the integrity of epithelial cells and contributed to their repair and regeneration. Our study has identified T- MSCs as a potential cell resource for IBD treatment.

## Introduction

Inflammatory bowel disease (IBD), including ulcerative colitis (UC) and Crohn's disease (CD), is characterized by recurrent chronic inflammation of the gastrointestinal (GI) tract. Multiple factors are involved in the onset and progression of IBD, including genetic predisposition, environmental factors, gut microbiota, and the immune system. Accumulative research has highlighted the essential role of host-microbiome interactions in IBD pathogenesis 1, especially the interplay between commensal microbiota and the gut immune system 2. Intestinal epithelium plays a key role in maintaining the homogeneity of the GI tract. Epithelial cells and their tight junctions form a physical barrier between luminal microbiota and lymphoid tissue. Specialized epithelial cells, such as goblet cells and Paneth cells, defend against bacteria through the secretion of mucus and α-defensins 3. Dysfunction of epithelial cells caused by inflammatory irritation or other injuries often results in the exposure of intestinal contents to the immune system and thus amplifies the inflammation. The integrity and regeneration potential of intestinal epithelial cells play a key role in defending against inflammation 4.

Mesenchymal stem cells (MSCs) refer to a heterogeneous population of spindle cells with the capacity to differentiate into osteoblasts, chondrocytes, and adipocytes *in vitro* 5. MSCs originate from a variety of tissues, including, bone marrow, umbilical cord, placenta, adipose and dental tissues. The therapeutic functions of MSCs lie in their immunomodulatory and trophic properties, making them a promising candidate for the treatment of autoimmune diseases and the development of regenerative medicine 6. In recent years, increasing evidence on the effectiveness of MSC-conditioned media or MSC-derived factors has provided insights about their trophic functions. MSCs secrete a series of mediators, including growth factors, cytokines, and chemokines, which promote tissue repair or reduce inflammatory responses 7. These bioactive factors possess multiple functions, such as modulating the local immune system, promoting angiogenesis, preventing cell apoptosis, and stimulating the survival, proliferation, and differentiation of resident tissue-specific cells 8.

Despite some promising results from clinical trials, MSC treatment for IBD is still in its infancy, with its underlying mechanisms still need to be explored in animal experiments and *in vitro* studies. Local MSC administration for perianal CD is a safe and effective method with increased healing rates and minor adverse effects. A phase III randomized controlled trial found that local injection of adipose-derived MSCs was more effective in closing external openings in CD patients with treatment-refractory complex perianal fistulas compared with placebos 9. However, the safety and efficiency of systemic MSC administration for luminal IBD remains unclear. Despite several studies reporting that MSC infusions contributed to colitis remission, a high heterogeneity existed among them, including tissue source, donator condition, culture and expansion methods, and the outcome measurement methods. Long-term observation and randomized, double-blind trials are still needed to make a more solid conclusion. To overcome some of the disadvantages of adult-tissue-derived MSCs, a new type of human embryonic stem cell (ESC)-derived MSCs, named as T-MSCs, has shown efficacy in some autoimmune disease models 10.

Multiple mechanisms are involved in the MSC treatment of IBD. MSCs' immunomodulatory characteristic is the most frequently mentioned property due to the immune dysfunction in the pathogenesis of IBD 6. MSCs interact with various types of cells in both innate and adaptive immune systems. Adipose-derived MSCs induced a distinct regulatory activation state of macrophages which possessed potent immunomodulatory abilities and protected mice from experimental colitis 11. MSC administration down-regulated both Th1-driven autoimmune responses and intestinal inflammation, observed by a decrease of a panel of inflammatory cytokines and an increase in regulatory IL-10 secretion 12. MSC treatment could also induce CD23+CD43+ regulatory B cells that contributed to alleviating intestinal inflammation 13. The key molecular mechanism for the therapeutic function lies in the secretion of anti-inflammatory factors, such as TNF-stimulated gene 6 (TSG6), Indoleamine 2,3-dioxygenase (IDO) 14, nitric oxide (NO) 15, prostaglandin E2 (PGE2) 16, and micro-RNAs 17. The interaction between MSCs and the tissue microenvironment of the inflammatory site is essential in the treatment stage 18.

Due to the heterogeneity of different MSC populations and the lack of knowledge in molecular mechanisms of embryonic stem cell-derived MSCs, we investigated their therapeutic efficacy in DSS-induced colitis in mice and explored the molecular basis for their therapeutic function.

## Methods

### MSC culture and preparation

Embryonic stem cell-derived MSCs (T-MSCs) were provided by ImStem Biotechnology Inc. All procedures followed the National Institute of Health's Guidelines on Human Stem Cell Research. ESI053 human embryonic stem cells were used in this study to derive T-MSCs. Phenotypic identification and tri-lineage differentiation were performed as previously reported 10.

MSCs were cultured in α-MEM (Gibco) supplemented with 10% human platelet lysate (Compass Biomedical), non-essential amino acids, and 2 mM L-glutamine. Culture media was changed every 48 h until cells reached 80% confluence. MSCs were then harvested by TrypLE (Gibco) and resuspended in PBS at 2×10^6^/mL for intravenous injection. P4 and P5 cells were used in this study, and phenotype markers (CD73, CD90, CD105) were examined by flow cytometry before animal experiments **(Figure S1A)**.

### Animal experiments and experimental design

All animal experiments were approved by the Southern Medical University Institutional Animal Care and Use Committee (K2018045). Male C57BL/6 mice (6-8 weeks) were obtained from Vital River Laboratory Animal Technology and housed in an SPF environment with free access to food and water.

In the acute DSS colitis model, mice were fed with 2.5% DSS (MP Biomedicals) in drinking water for 5 consecutive days. The T-MSC group received a single injection of 5×10^5^ cells in 250 μL PBS on day 3 through the tail vein, and the DSS group received the same volume of PBS. The body weight and disease activity index (DAI) score of each mouse were recorded daily according to standard protocols 19. Mice were sacrificed on day 9 and samples, including the serum, colon, and liver, were harvested. Mouse High Sensitivity T Cell Magnetic Bead Panel kit (MILLIPLEX® MAP) was used to measure serum cytokines according to the manufacturer's instructions. Hematoxylin-Eosin (HE) staining of colon slides was performed, and histopathology activity index (HAI) scores were evaluated. Scoring systems for DAI and HAI are listed in supplementary materials **(Table S1)**.

In the chronic DSS colitis model, a cycle was defined as a 5-day oral administration of 1.5% DSS followed by a 10-day administration of water. The entire experiment included 3 cycles during which body weight and DAI scores were evaluated daily. The T-MSC group received 5×10^5^ intravenous cell administration on days 3, 18, and 33, while DSS group received PBS. Mice were sacrificed on day 39, serum and colon samples were harvested.

For the IGF-1 receptor inhibitor experiment, acute DSS colitis was induced and two IGF-1 receptor inhibitors Linsitinib (OSI906, Selleck) and Picropodophyllin (PPP, Selleck) were applied. The OSI906 and PPP groups each received daily intraperitoneal administration of 30 mg/kg of respective inhibitors and a single T-MSC injection of 5×10^5^ cells. The IGF-1 group received an intravenous injection of 2 mg/kg recombinant mouse IGF-1 (R&D systems) instead of a T-MSC administration. DSS, T-MSC, and IGF-1 groups all received daily intraperitoneal administrations of inhibitor vehicles. Mice were sacrificed on day 9, and colon, intestine, liver, and serum samples were harvested.

### *In vivo* tracking of T-MSCs

For the Dil labeling of T-MSCs, cells were incubated with 5 μL/mL CM-DiI (Molecular Probes) at 37 ºC for 30 min, and the labeling efficiency was measured by flow cytometry and fluorescence microscopy **(Figure S2A)**. For GFP and luciferase labeling of T-MSCs, cells were transfected with GFP and luciferase-labeled lentiviral vector (Umine-bio) and observed under a microscope **(Figure S2A)**. Mice were fed with 2.5% DSS and labeled T-MSCs were administrated intravenously or intraperitoneally on day 3. *In vivo* imaging of luciferase was performed by injecting 200 μL luciferase substrate (15 g/L, Sigma) intraperitoneally, and mice were photographed using Bruker *In vivo* FX Pro. Mice were then sacrificed at specific time points. Immunofluorescence stains of CD 31 (Abcam) and F4/80 (CST) were performed according to the manufacturer's instructions. Cryosections of different organs were then photographed by a confocal microscope (FV 1000, Olympus).

### Epithelial barrier permeability measurement

Mice intestinal barrier permeability was measured as previously reported 20. In brief, mice were gavaged with 150 μL of 4 kDa fluorescein isothiocyanate (FITC)-dextran (Sigma) 4 h before sacrifice. Serum FITC-dextran fluorescence was detected by a plate reader at 485 excitation/528 emission and concentrations were calculated according to the standard curve.

### BrdU incorporation assay and immunohistochemical staining

For the *in vivo* BrdU incorporation assay, 100 μL of 10 mg/mL BrdU solution (Sigma) was injected intraperitoneally. Mice were sacrificed 4 h after the injection, and colon tissues were harvested.

For immunohistochemical staining of BrdU, Ki-67, Bmi1, TERT, and IGF-1 in samples of acute DSS colitis model, tissues were fixed by 4% paraformaldehyde and embedded in paraffin. Tissues were then sliced into 4 µm-thick sections, and antigens were retrieved using citrate solution. Anti-Ki-67, -IGF-1, -Bmi1, -TERT (Abcam), and -BrdU (CST) were used as primary antibodies and incubated at 4 ºC overnight. Goat-anti-rabbit IgG was used as a secondary antibody and diaminobenzidine (DAB) staining was performed. Sections were scanned using the Aperio ScanScope CS System and positive rates were calculated by Image J software (National Institutes of Health).

### RNA isolation and Real-Time PCR

Liver RNA was isolated using a column-based isolation kit (EZbioscience) according to the manufacturer's instructions and the concentrations were measured by a spectrophotometer (Nanodrop 2000, Thermofisher). Equal amounts of RNA (1 μg) were used to generate cDNA through reverse transcription and real time-qPCR was performed to measure the expression of Igf-1 and Igfbps (Takara). RT-PCR primer sequences are listed in **Table S2**.

### ELISA for serum and tissue IGF-1

Whole blood samples were centrifuged at 4 ºC 2500 rpm for 20 min and serum was collected. Tissue samples (colon, intestine, and liver) were homogenized in a lysis buffer (RIPA with protease inhibitor, Beyotime) and whole protein concentrations were measured by Bicinchoninic Acid assay. IGF-1 duo-set ELISA kit (R&D Systems) was used to measure IGF-1 concentrations according to the manufacturer's protocols. Results were read by a microplate reader (SpectraMax190, Molecular Devices), and concentrations were calculated by SoftMax Pro 5.0.

### Colonic epithelial cell protein isolation and Western blotting

Colon epithelium isolation was performed according to the previously reported method 21. In brief, colon tissues were washed thoroughly in cold PBS and contents were flushed away. Colons were then cut into 5 mm pieces and put into a digestion buffer (5 mM EDTA and 2 mM DTT in Hanks balanced salt solution, Sigma) and incubated at 37 ºC for 30 min on a rotating platform. Equal volumes of HBSS were added to stop digestion and the solution was filtered through a 70 μm cell strainer. The filtered solution was centrifuged at 4 ºC 2000 rpm for 15 min to obtain epithelial cell pellets for protein isolation.

Western blotting was performed as described in a previously reported protocol 22. GAPDH, p44/42 MAPK, phospho-p44/42 MAPK, AKT (pan), phospho-AKT (Ser473), IGF-1 Receptor β, phospho-IGF-1 Receptor β (Tyr1131), mTOR, phospho-mTOR (Ser2448), PI3 Kinase p110α, and p70s6 Kinase rabbit antibodies were all from CST. Total protein and epithelial protein expressions of colons were measured.

### Colonic mucosa sample collection from IBD patients

The sample collection procedure was approved by the Southern Medical University Ethics Committee (NFEC-2014-040) and patients were provided with informed consent. Patients diagnosed with UC or CD were included and colonic mucosa of the inflammatory site and its adjacent normal control were collected as a sample pair during colonoscopy. Sample pairs were preserved in liquid nitrogen for further analysis.

### *In vitro* cell function assay

Human colon epithelial cell lines NCM 460 and FHC were used in this study. Cells were cultured in DMEM (Gibco) supplemented with 10% serum (Hyclone) for routine cultures and in DMEM with 0.5% serum for cell function experiments. To evaluate the cell proliferative potency, cells were seeded in a 96-well-plate at a density of 3000 cells/well and a Cell Counting Kit-8 (CCK-8, Dojindo) assay was performed. OD 450 values were measured every 24 h for 6 days. Cell apoptosis was induced by 50 ng/mL TNF-α (Peprotech) for 3 days. Annexin V and 7-AAD staining (BD bioscience) was performed, and cells were tested by a flow cytometer (Aria III, BD bioscience). Cell cycle analysis was measured by *in vitro* BrdU incorporation and performed following the manufacturer's instructions (MultiSciences). BrdU-incorporated cells were measured by flow cytometry. Data were analyzed by Flowjo 10.0 software.

### Co-culture of T-MSCs and mice primary hepatocytes

Hepatocytes of colitis mice (2.5% DSS for 3 days) were isolated as previously described 23. Briefly, the liver was perfused with 1 mg/mL type IV collagenase (Sigma) in DMEM/F12 supplemented with 2% FBS. Isolated hepatocytes were cultured in rat tail collagen-treated 12-well-plates in DMEM/F12 with 20% FBS. T-MSCs were added 24 h after the isolation and co-cultured with hepatocytes in both cell-cell contact and Transwell systems. Supernatant IGF-1 was measured by ELISA 48 h after the co-culture.

### Mouse colon organoid culture and immunofluorescence staining

Mouse colon organoid isolation was performed according to the manufacturer's protocols. In brief, the colon was harvested and luminal contents were flushed away by cold PBS in a 10 cm dish. The colon was then transferred to a biosafety cabinet and cut into 2-5 mm pieces. Colon pieces were washed thoroughly in cold PBS, and crypts were digested using Gentle Cell Disassociation Reagent (StemCell Technologies) on a rocking platform for 20 min. The supernatant was removed, and colon pieces were resuspended in cold PBS with 0.1% BSA. The new supernatant was then passed through a 70 μm strainer and centrifuged at 4 ºC 290×g for 5 min to obtain crypts. Isolated crypts were then resuspended in DMEM/F12 with 15 mM HEPES (StemCell Technologies) and counted under a microscope. An equal volume of IntestiCult Organoid Growth Medium (StemCell Technologies) and Matrigel (Corning) were mixed and the suspension was transferred into a pre-warmed 24-well-plate to form domes in the middle of each well. Complete IntestiCult Organoid Growth Medium was added above the domes, and crypts were cultured at 37 ºC and 5% CO_2_.

For the PCNA staining, colon organoids were fixed by paraformaldehyde and incubated with anti-PCNA antibody (CST) at 4 ºC overnight and goat-anti-rabbit IgG (FITC Conjugate, Beyotime) at room temperature for 1 h. Organoids were then observed under a fluorescence microscope (IX73, Olympus).

### Serum cytokine antibody array

Serum samples from the acute colitis model were collected and G-Series Mouse Cytokine Antibody Array 4000 (GSM-CAA-4000) was used to measure the relative levels of 200 cytokines **(Table S3)** according to protocols in the user manual. Fluorescence signals were detected by Axon GenePix, and the relative level of cytokines was calculated and analyzed.

### RNA sequencing

Colons were harvested and RNA samples were isolated as previously described. RNA quality control tests, including concentration, purity, integrity, and contamination evaluation, were performed to ensure that the quality was maintained for subsequent procedures.

A total amount of 3 µg RNA per sample was used as the input material for the RNA sample preparation. Sequencing libraries were generated using NEBNext UltraTM RNA Library Prep Kit from Illumina (NEB), following the manufacturer's recommendations, and index codes were added to attribute sequences to each sample.

The clustering of the index-coded samples was performed on a cBot Cluster Generation System using the TruSeq PE Cluster Kit v3-cBot-HS (Illumia) according to the manufacturer's instructions. After cluster generation, the library preparations were sequenced on an Illumina Hiseq platform and 125 bp/150 bp paired-end reads were generated.

Raw data of fastq format were first processed through in-house Perl scripts. In this step, clean data were obtained by removing reads containing adapter and trimming low-quality bases with Trimmomatic.

Reference genome and gene model annotation files were downloaded from the ensemble database. The reference genome index was built using Hisat2 v2.0.5 and paired-end clean reads were aligned to the reference genome using Hisat 2 v2.0.5. Htseq-count was used to count the read numbers mapped to each gene. Subsequently, FPKM of each gene was calculated based on the length of the gene and reads count mapped to this gene. Differential expression analysis of DSS and T-MSC group was performed using the DESeq2 R package (1.16.1). Differentially expressed genes (DEGs) were defined as FDR<0.05 and FC>2. Gene Ontology (GO) enrichment analysis of differentially expressed genes was implemented by the topGO R package, in which gene length bias was corrected. Cluster Profiler R package was used to test the statistical enrichment of differential expression genes in the Kyoto Encyclopedia of Genes and Genomes (KEGG) pathways. Protein-protein interaction (PPI) analysis of up-regulated DEGs was performed using the String database.

### Statistical analysis

Statistical analysis was performed by GraphPad Prism 8.0 (GraphPad Software). All variables were expressed in mean ± SD format with at least 3 replicates in each group. Analysis of variance for repeated measurements (RM-ANOVA) was used to compare body weights and OD450 values. Student *t*-test was used to compare 2 groups. One-way ANOVA and Tukey's analysis were used when comparing 3 or more groups. Mann-Whitney test and Kruskal-Wallis test were used for comparing nonparametric data. Statistical significance was defined as *p*<0.05.

## Results

### Intravenous T-MSC treatment was therapeutic in the DSS colitis model in mice

**Figure 1:**
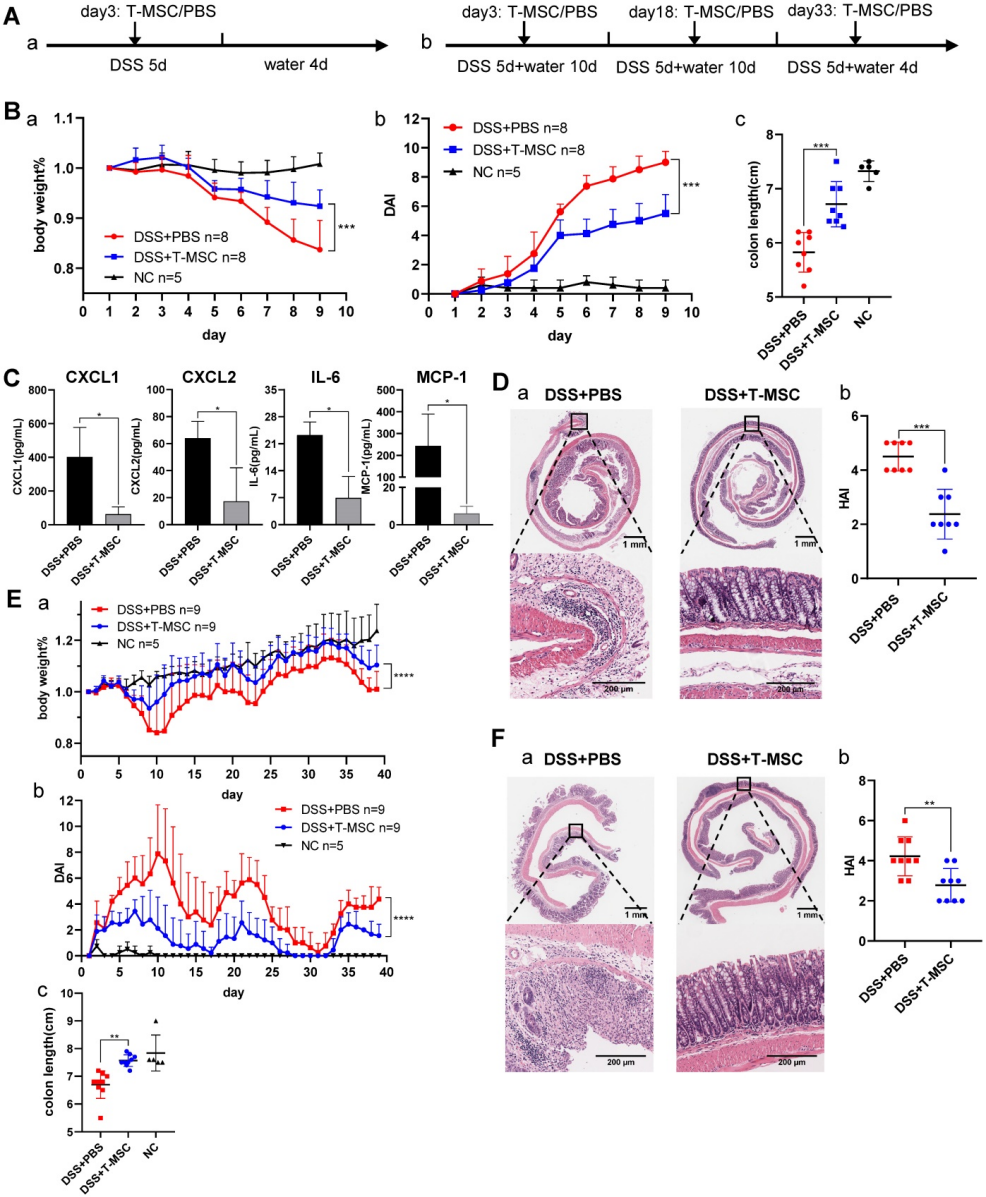
** Intravenous T-MSC administration alleviated DSS-induced colitis in mice. A.** Experimental layout of T-MSC treatment in the acute and chronic DSS colitis. The acute colitis model was induced by 2.5% DSS in drinking water for 5 consecutive days. Mice received an intravenous injection of 5×10^5^ T-MSCs (n=8) or PBS (n=8) on day 3. The chronic colitis model was induced by 3 cycles of 1.5% DSS (5 d) + water (10 d or 4 d). Mice received intravenous injections of 5×10^5^ T-MSCs (n=9) or PBS (n=9) on day 3, 18, and 33. Negative control (NC) group mice were maintained on untreated drinking water (n=5 in both acute and chronic DSS colitis models). **B.** Therapeutic efficacy evaluation of acute DSS model. a. bodyweight percentage. b. DAI scores. c. colon length. **C.** Serum inflammatory cytokines in acute DSS colitis model measured by mouse high sensitivity T cell magnetic bead panel. **D.** a. HE staining of colon sections in the acute colitis model. Scale bar = 1 mm top panel/200 µm bottom panel. b. HAI scores of colons. **E.** Therapeutic efficacy evaluation of chronic DSS model. a. bodyweight percentage. b. DAI scores. c. colon length. **F.** a. HE staining of colon sections in the chronic colitis model. Scale bar = 1 mm top panel/200 µm bottom panel. b. HAI scores of colons. Data are expressed as mean±SD. *p < 0.05, **p < 0.01, ***p < 0.001 and ****p < 0.0001. DAI: disease activity index; HAI: histopathology activity index.

The colitis mouse models were established as described in the Methods section and depicted in **Figure 1A**. First, an acute DSS colitis model was induced, and T-MSCs were injected intravenously. Compared to the DSS+PBS group, the DSS+T-MSC group showed reduced body weight loss and lower DAI scores **(Figure 1B)**. The average colon length was reduced to a lesser degree in the T-MSC group than PBS controls (**Figure 1B, S1B**). Serum samples were collected and MILLIPLEX MAP mice T Cell Magnetic Bead Panel was used to detect various cytokines. Among the measured cytokines, CXCL1, CXCL2, IL-6, and MCP-1 were significantly reduced in the T-MSC group **(Figure 1C)**. Other inflammatory cytokines were also reduced in the T-MSC group but failed to reach statistical significance **(Figure S1C)**. Colons were cut along the vertical axis and rolled into an onion shape to increase the length for histopathology evaluations. HE sections indicated that epithelium loss and inflammatory cell infiltration were significantly reduced in the T-MSC group and HAI scores were also reduced after T-MSC injection **(Figure 1D)**.

Chronic DSS colitis was induced by 3 cycles of DSS + water combinations and intravenous T-MSC injection was performed on day 3 of DSS administration in each cycle. Similar to the acute colitis model, the T-MSC group showed reduced body weight loss patterns and lower DAI scores **(Figure 1E)**. T-MSC group's colon tissues showed less erosion and fewer lymphocyte infiltrations in the epithelium and submucosa layer** (Figure 1F)**. The HAI scores were also decreased in the T-MSC group **(Figure 1F)**.

Several treatment methods including local injections 11 and systemic administrations 24 were reported for mouse colitis MSC therapy. We found that a single intraperitoneal injection of T-MSCs had limited therapeutic effects compared with intravenous administration with no significant difference in body weight and colon length between the intraperitoneal group and PBS controls. However, some serum inflammatory cytokines (TNF-α, IL-6) were considerably reduced in the intraperitoneal group (**Figure S1D**). The inflammatory microenvironment has been reported to play a key role in evoking MSC's therapeutic potential 25. However, we observed that *in vitro* TNF-α or IFN-γ treatment diminished T-MSCs' therapeutic efficacy (**Figure S1E**). This indicated that different molecular mechanisms might be involved with* in vitro* inflammatory cytokine stimulation and *in vivo* inflammatory microenvironment. The therapeutic potential of another human embryonic stem cell line, H9-derived MSCs, was evaluated for the treatment of DSS colitis and compared with ESI053-derived T-MSCs. Both cell types showed similar therapeutic efficacy in treating acute DSS colitis in mice (**Figure S1F**).

### Intravenously injected T-MSCs were mainly distributed in the lungs, liver, and spleen

**Figure 2:**
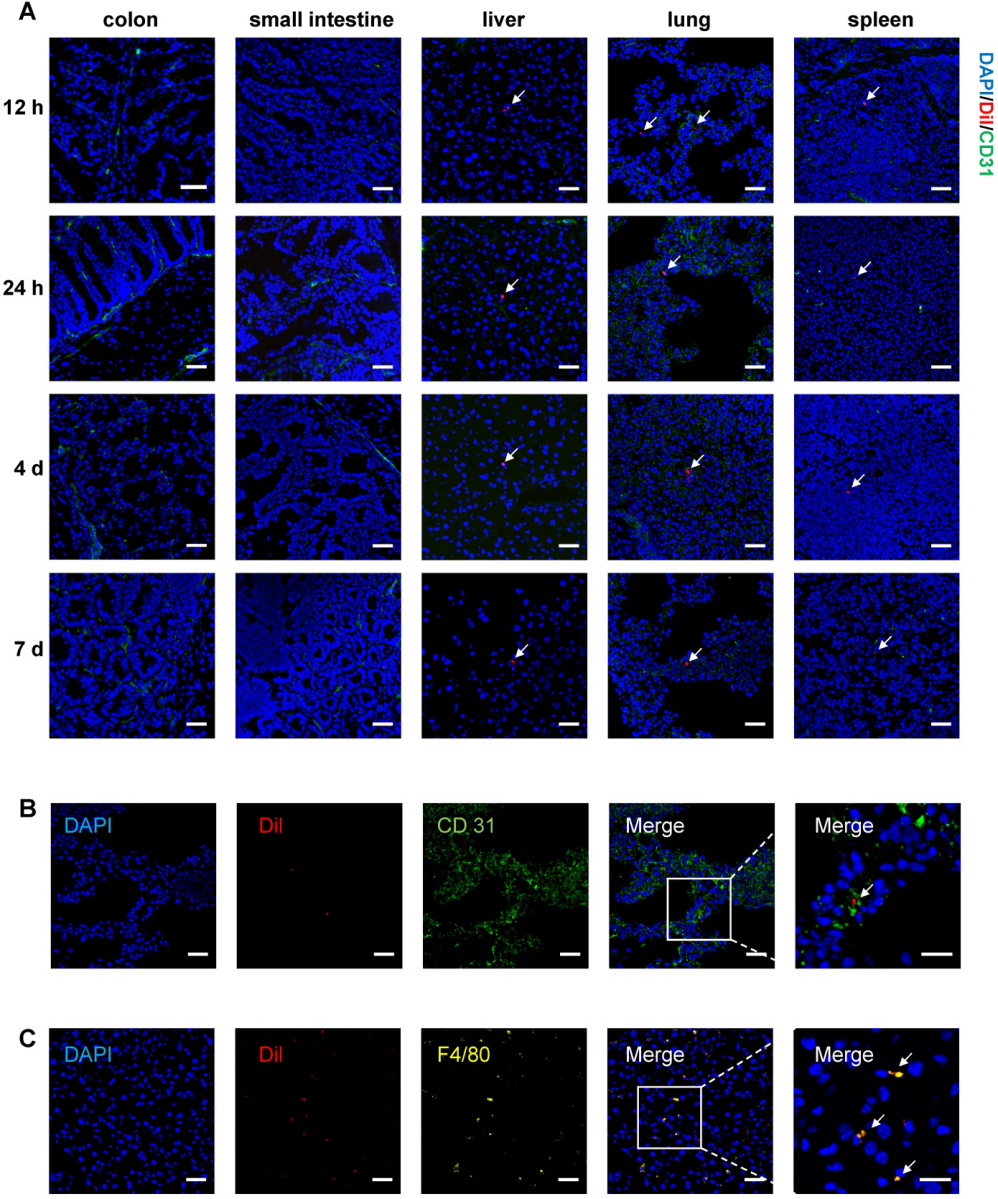
***In vivo* tracking of Dil-labeled T-MSC. A.** Mice were fed with 2.5% DSS for 3 days, and Dil-labeled T-MSCs were injected intravenously. *In vivo* distribution of T-MSCs was observed by immunofluorescence staining of the colon, small intestine, liver, lung, and spleen. Scale bar = 50 µm **B.** Immunofluorescence staining of the vascular endothelial cell marker CD 31 in frozen lung sections. Scale bar = 50 µm (left panel)/20 µm (right panel, magnified view). **C.** Immunofluorescence staining of the macrophage marker F4/80 in frozen sections of the liver. Scale bar = 50 µm (left panel)/20 µm (right panel, magnified view). Arrows indicate labeled cell components.

To track T-MSCs after injection, cells were labeled with CM-Dil for long-term observation and injected intravenously or intraperitoneally on day 3 of DSS administration. Mice were sacrificed at 12 h, 24 h, 4 d, and 7 d after the injection and cryosections of multiple organs were analyzed. For intravenous injection, labeled cells were mainly distributed in the lungs and a small proportion of cells could be observed in the liver and spleen. Fluorescent signals of Dil-labeled T-MSCs could be detected 12 h after injection and weakened signals could still be observed 7 days after injection (**Figure 2A**). Dil-labeled T-MSCs were not observed in the colon and small intestine after intravenous administration, indicating that injected cells failed to migrate to inflammatory colon tissues** (Figure 2A)**. Besides, intraperitoneally injected T-MSCs were not observed in any organ during any time interval **(Figure S2B)**.

To further confirm the distribution of injected T-MSCs, cells were transfected with GFP and luciferase-labeled virus and injected intravenously or intraperitoneally. Similar to Dil-labeled T-MSCs, GFP-labeled T-MSCs could be observed in the lungs, liver, and spleen 12 h and 24 h after intravenous administration **(Figure S2C)**. Interestingly, luciferase-expressing T-MSCs could be detected near the injection site 12 and 24 h after intraperitoneal administration. However, the signal was not detected after intravenous injection **(Figure S2D)**, indicating that most of the intravenously administered T-MSCs were removed from the injection site via blood circulation, and the cell number at the targeted organs was too small for bioluminescence imaging**.** It was also possible that the cells were not alive after intravenous injection.

To specify T-MSCs' location in the lungs and liver, immunofluorescence staining of vascular endothelial cell marker CD 31 and macrophage marker F4/80 was performed. We observed T-MSCs near endothelial cells in the lungs, indicating that T-MSCs might be blocked in the lung vessels (**Figure 2B**). In the liver, T-MSCs showed co-location with F4/80 fluorescence, which could be due to macrophages engulfing the injected cells as a foreign component (**Figure 2C**).

MSC tracking experiments indicated that in the colitis mouse model, intravenously injected T-MSCs did not migrate to the inflammatory site. Instead, systemically administrated cells were mainly distributed in the lungs, liver, and spleen, indicating that injected cells might interact with tissue-resident macrophages or other cell types in circulating blood.

### T-MSC treatment increased serum IGF-1 level

**Figure 3:**
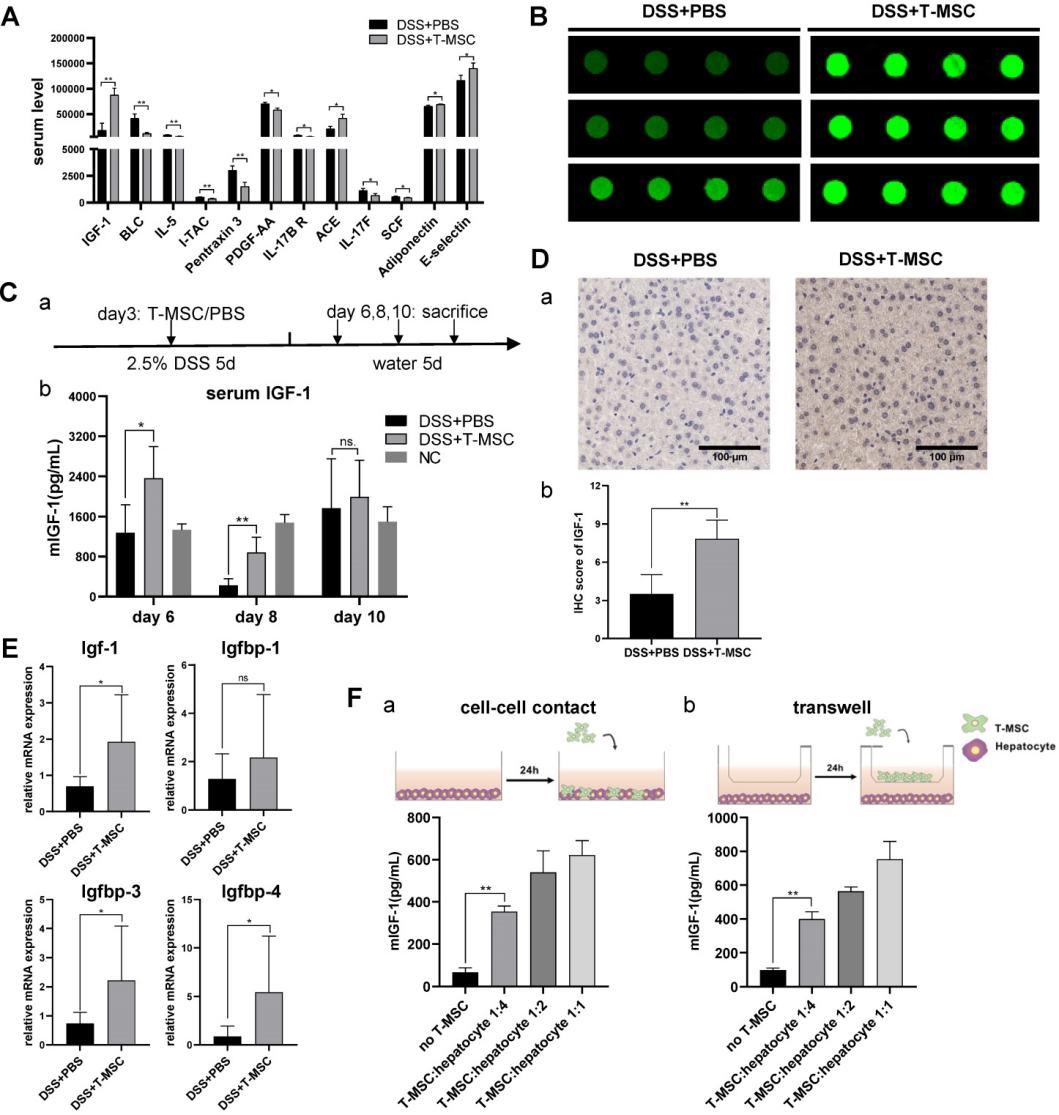
** T-MSC treatment increased serum IGF-1 level. A.** Serum cytokines of T-MSC-treated mice and PBS controls in acute colitis model were measured by the antibody array test. Normalized signal values were analyzed by student t-test, cytokines with *p*<0.05, and signal value>500 are listed. **B.** Fluorescent signals of IGF-1 detected by a microarray scanner in the antibody array test. **C.** Serum IGF-1 expression was verified by ELISA. a. experimental layout of the T-MSC treatment model. b. mice serum samples were collected on day 6, day 8, and day 10, and IGF-1 levels were detected by ELISA. **D.** Immunohistochemical staining of IGF-1 in the liver. a. IHC staining of liver sections. b. IHC scores of IGF-1. Scale bar = 100 µm. **E.** RT-qPCR for relative mRNA expression (normalized to GAPDH) of Igf-1 and Igfbps (Igfbp1, Igfbp3, Igfbp4) in the liver. **F.** T-MSCs and mouse primary hepatocytes were co-cultured at different ratios in cell-cell contact or Transwell assays, and supernatant IGF-1 was measured. Data are expressed as mean ± SD. *p < 0.05 and **p < 0.01. mIGF-1: mouse IGF-1.

Since intravenously injected MSCs failed to migrate to the colon tissue, we speculated that their therapeutic potential might be realized either by interactions with other cells in the circulatory system or through paracrine functions. To test these possibilities, we collected serum samples from acute colitis model and screened for potential therapeutic proteins using antibody arrays for 200 serum cytokines and identified 12 cytokines with statistically significant differences (*p*<0.05, signal value>500) (**Figure 3A**). Among the 12 cytokines, mouse IGF-1, reported to function in promoting repair and regeneration in other disease models 26, showed the highest-fold change in fluorescence signals **(Figure 3B)**. Nevertheless, its binding protein levels were similar between the T-MSC treatment and PBS control groups (**Figure S3A**). Since angiogenesis and trophic functions are involved in MSCs' therapeutic mechanisms, representative cytokines were tested but showed no difference between the two groups (**Figure S3B**).

We further analyzed serum IGF-1 and observed higher levels in the T-MSC treatment model than in controls on day 6 and day 8 (**Figure 3C**). This indicated that the IGF-1 level was increased in an early repair stage of the DSS colitis model and might play a role in mucosal regeneration. The origin of mouse serum IGF-1 is not clear as it is secreted by multiple organs and various cell types. The main source of IGF-1 is the liver with hepatocytes secreting approximately 80% of the circulating IGF-1 27.

We performed IHC staining of the liver and detected increased IGF-1 expression in the T-MSC group compared to PBS controls (**Figure 3D**). The binding of IGF-1 to its binding proteins (IGFBPs) is known to extend its half-life. IGFBP-3 is the most abundant while IGFBP-2 and IGFBP-5 exert inhibitory functions. RT-PCR analysis of liver tissues was performed and increased mRNA expression of Igf-1, Igfbp3 and Igfbp4 was observed in the T-MSC group (**Figure 3E**). Hepatocytes of colitis mice were isolated and co-cultured with T-MSCs at ratios of 1:1, 1:2, and 1:4 (T-MSCs: hepatocytes). Mouse IGF-1 of the supernatant was measured and found to be increased in both cell-cell contact and Transwell assays, indicating that T-MSCs promoted IGF-1 secretion from hepatocytes in cell contact-dependent or -independent manner (**Figure 3F**). To further confirm the origin of IGF-1, T-MSCs were transfected with siRNA to down-regulate the expression of Igf-1 and the therapeutic efficacy was re-evaluated in an acute DSS colitis model. Both si-Igf1 and si-NC groups showed similar therapeutic functions compared to the T-MSC group (**Figure S3C-F**). These results suggested that elevated circulating IGF-1 in the T-MSC group was likely derived from the endogenous secretion of liver.

### IGF-1 receptor inhibitors blocked T-MSCs' therapeutic efficacy

**Figure 4:**
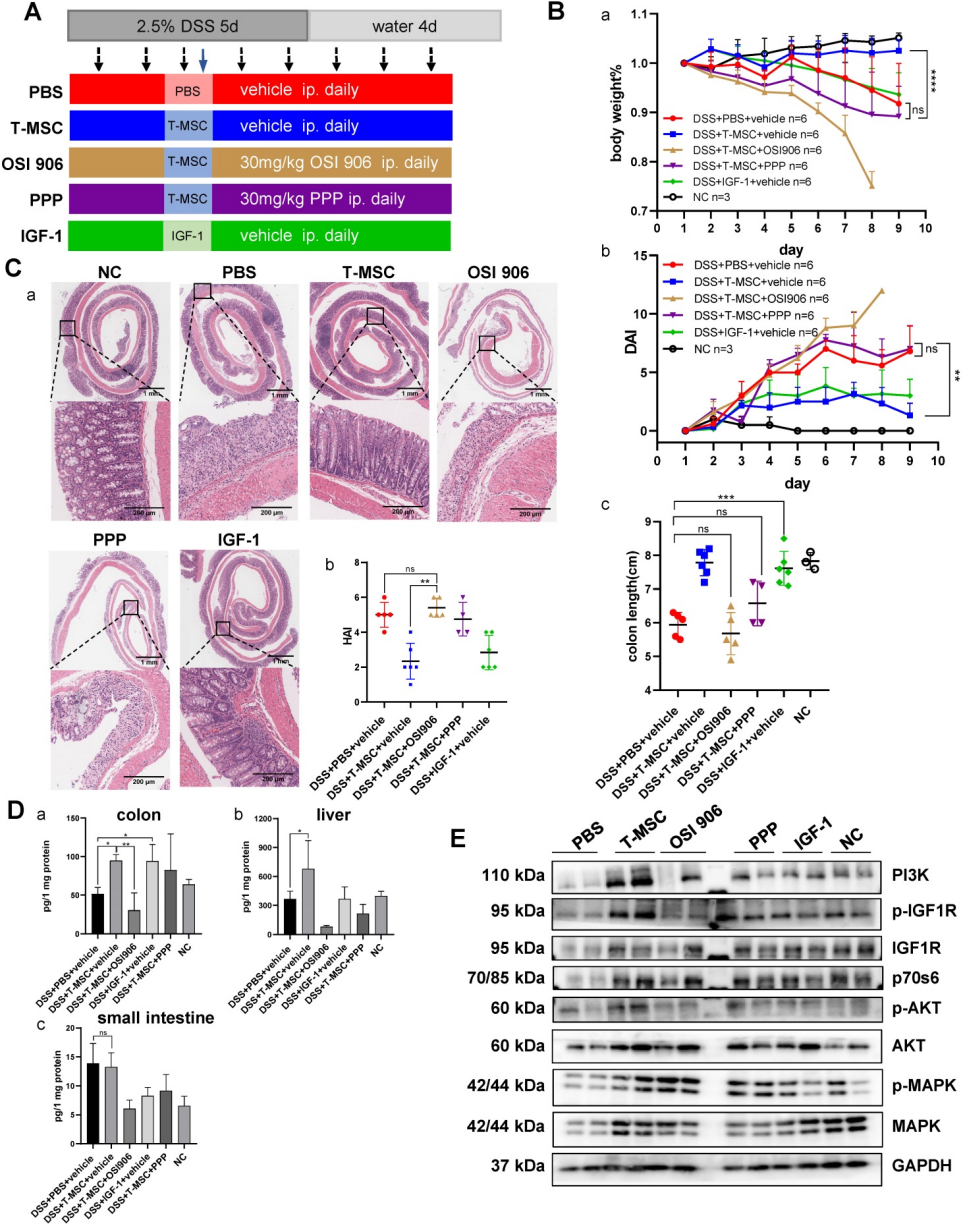
** IGF-1 receptor inhibitors blocked the therapeutic efficacy of T-MSC administration. A.** Experimental scheme for the IGF-1 receptor inhibitor model. Colitis was induced by 2.5% DSS in drinking water for 5 consecutive days. DSS + PBS + vehicle group received daily intraperitoneal injections of inhibitor vehicles for 8 consecutive days and a single intravenous injection of PBS on day 3. DSS + T-MSC + vehicle group received daily intraperitoneal injections of inhibitor vehicles for 8 consecutive days and a single intravenous injection of 5×10^5^ cells on day 3. DSS + T-MSC + OSI 906 group received daily intraperitoneal injections of 30 mg/kg OSI 906 for 8 consecutive days and a single intravenous injection of 5×10^5^ cells on day 3. DSS + T-MSC + PPP group received daily intraperitoneal injections of 30 mg/kg PPP for 8 consecutive days and a single intravenous injection of 5×10^5^ cells on day 3. DSS + IGF-1 + vehicle group received daily intraperitoneal injections of inhibitor vehicles for 8 consecutive days and a single intravenous injection 2 mg/kg IGF-1 on day 3. **B.** Therapeutic efficacy was evaluated in each group. a. bodyweight percentage. b. DAI scores. c. colon length. **C.** a. HE staining of colon sections in different groups. Scale bar = 1 mm (top panel)/200 µm (bottom panel). b. HAI scores of colon sections. **D.** ELISA for normalized IGF-1 levels in different tissues. a. IGF-1 expression in the colon. b. IGF-1 expression in the liver. c. IGF-1 expression in the small intestine. **E.** IGF1-IGF1R downstream protein expressions were measured by Western blotting. Immunoblotting of MAPK, p-MAPK, AKT, p-AKT, IGF1R, p-IGF1R, p70s6, and PI3K were performed. Data are expressed as mean ± SD. *p < 0.05, **p < 0.01, ***p < 0.001 and ****p < 0.0001. DAI: disease activity index; HAI: histopathology activity index.

To confirm the role of IGF-1 in T-MSC treatment efficacy of DSS colitis, T-MSC-treated mice received daily intraperitoneal injections of IGF-1 receptor inhibitors OSI906 and PPP for 8 consecutive days and the therapeutic efficacy was evaluated (**Figure 4A**). Despite receiving T-MSC injections, colitis significantly worsened in inhibitor groups. Reduced body weight, shortened colon length, and higher DAI scores were observed (**Figure 4B, S4A**), and HE sections also showed aggravated colon inflammation in the inhibitor groups (**Figure 4C**). Intravenous injection of 2 mg/kg recombinant mouse IGF-1 increased the colon length but failed to decrease the body weight loss and DAI scores. Due to the significant weight loss and colon inflammation presented in inhibitor groups, especially in the OSI 906 group, untreated healthy mice received the same intraperitoneal injection as the inhibitor groups and body weight was recorded for 9 days to exclude the influence of inhibitor administration on the body weight (**Figure S4C**). We also compared the colitis between DSS+T-MSC+inhibitor and DSS+PBS+inhibitor groups and found that T-MSC injection failed to improve the worsened colitis in the inhibitor group (**Figure S5**).

To further determine the role of IGF-1, different doses (2 mg/kg and 4 mg/kg) and injection methods (intravenous and intraperitoneal) were applied and compared with the T-MSC group. Intravenous injection of 4 mg/kg IGF-1 improved the body weight and DAI scores compared with other IGF-1 groups but was still less effective than the T-MSC group as per HE staining of colon sections (**Figure S6**). This indicated IGF-1 plays an important role in the treatment, while other mechanisms may also work together to reduce colitis.

Normalized IGF-1 from different tissues was measured by ELISA. The T-MSC group showed increased IGF-1 expression in liver and colon tissues compared with the control and inhibitor groups. However, this trend was not observed in intestinal tissues (**Figure 4D**). This further proved that IGF-1 was increased in the inflammatory site and was likely originated from the liver.

The IGF-1 receptor is a tyrosine kinase receptor expressed in most cell types 28. Binding of IGF-1 to its receptor leads to phosphatidylinositol 3-kinase (PI3K) recruitment, promoting the expression and phosphorylation of AKT 29. Mammalian target of rapamycin (mTOR) and mitogen-activated protein kinase (MAPK) are also involved in downstream signal transduction of IGF-1. Western blotting of colon proteins showed elevated expressions of MAPK, p-MAPK, AKT, p-AKT, IGF1R, p-IGF1R, PI3K, and p70s6 in the T-MSC group compared to PBS and inhibitor groups (**Figure 4E, S4E**). The levels of IGF1R, AKT, and their phosphorylated forms were all increased. This suggested that the IGF1R-PI3K-AKT pathway in the colon was activated after T-MSC treatment. mTOR and p-mTOR expression showed no apparent difference among various groups (**Figure S4D**).

### MSCs improve colon epithelial proliferation and integrity via increased IGF-1

**Figure 5:**
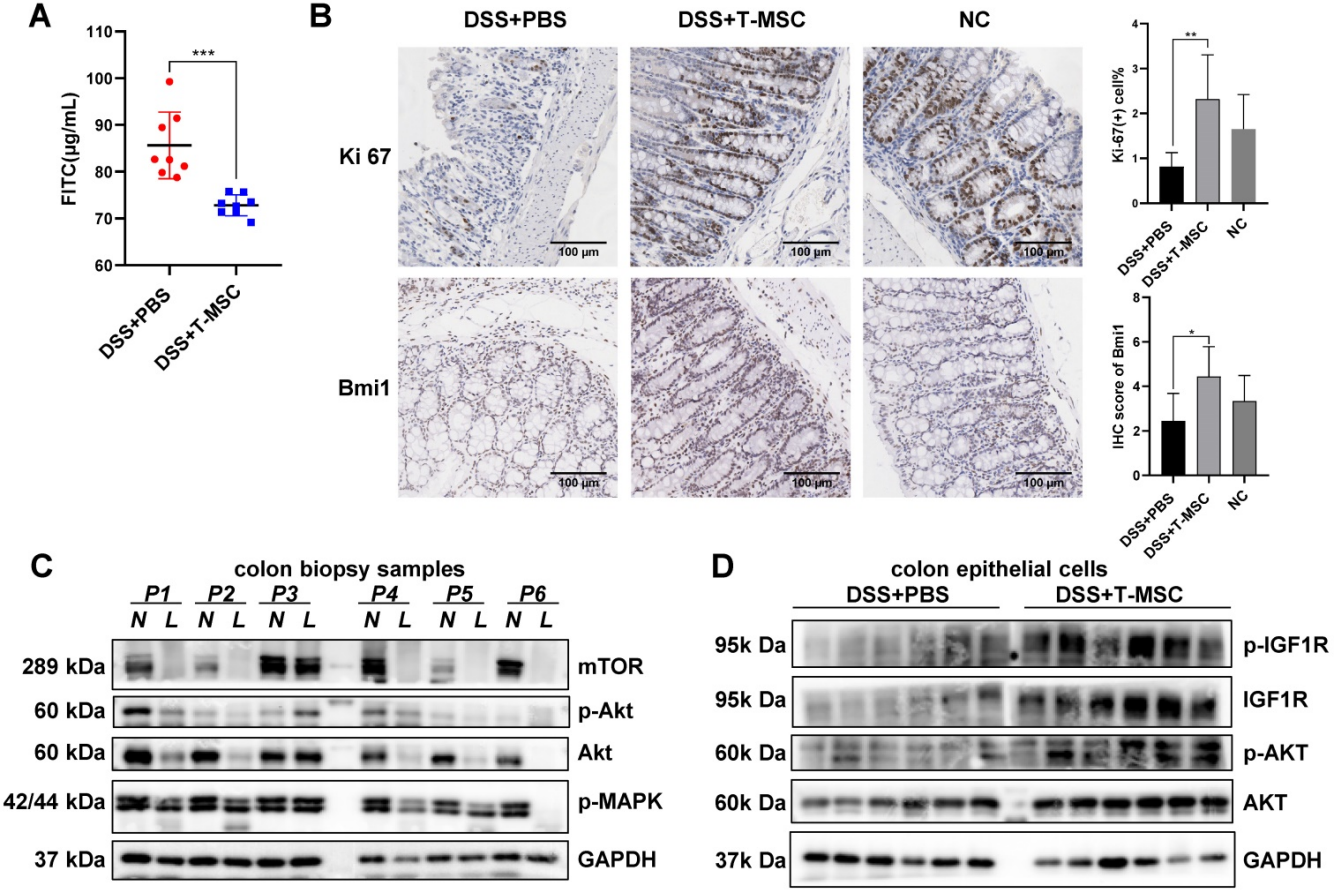
** T-MSC treatment maintained proliferative potency of colon epithelium. A.** FITC-dextran solutions were administered intra-gastrically, and serum FITC concentration in the acute colitis model was detected 4 h after administration. **B.** The proliferative potential of the remaining colon epithelium in the acute colitis model was measured by IHC staining of Ki-67 and intestinal stem cell marker Bmi1. Scale bar = 100 µm. **C.** Inflammatory mucosae (L, lesion) and its adjacent normal mucosae (N, normal) of IBD patients were collected and expressions of p-MAPK, AKT, p-AKT, and mTOR were measured by Western blotting. **D.** Colon epithelial cells in the acute colitis model were isolated and expressions of AKT, p-AKT, IGF1R, and p-IGF1R were measured by Western blotting. Data are expressed as mean ± SD. *p < 0.05, **p < 0.01, ***p < 0.001.

IGF-1 is involved in multiple biological processes, including cell growth, proliferation, apoptosis, and cell cycle, critical in regulating anabolic activities and tissue repair in various organs. The regeneration potency of colonic mucosa is a key factor in defending the damage and inflammation caused by various pathogenic processes. The integrity of crypt cells and epithelial barriers are basic elements in maintaining the proliferative ability of epithelium. T-MSC treatment maintained the integrity of colonic mucosa by reducing epithelial barrier permeability measured by serum FITC-dextran concentration (**Figure 5A**). The remaining epithelial crypts in the treatment group also showed greater proliferation potential than the PBS controls, characterized by an increase in Ki-67 positive cells and BrdU incorporation (**Figure 5B, S7A**). IHC staining of colonic stem cell markers Bmi1 and TERT showed that the expression of Bmi1 was increased in the T-MSC group while TERT showed no difference (**Figure 5B, S7A**), implying that T-MSC injection contributed to the regenerative potential of colonic epithelium.

The downstream pathway of IGF-1 might be involved in the inflammation and repair of the intestinal epithelium. Immunoblotting analysis of colonic mucosae from IBD patients showed impaired expression of AKT and mTOR in the inflammatory site compared with the adjacent normal epithelium while the expression of IGF1R showed no difference (**Figure 5C, Figure S7B**). Protein analysis of mouse isolated colonic epithelial cells showed increased expression of IGF1R, p-IGF1R, AKT, and p-AKT in the treatment group (**Figure 5D**). IGF1R, AKT, and their phosphorylated forms showed increased protein expression after T-MSC injection, while the expression of MAPK and mTOR showed no difference (**Figure S7C**).

### Transcriptome analysis of colon tissues

**Figure 6:**
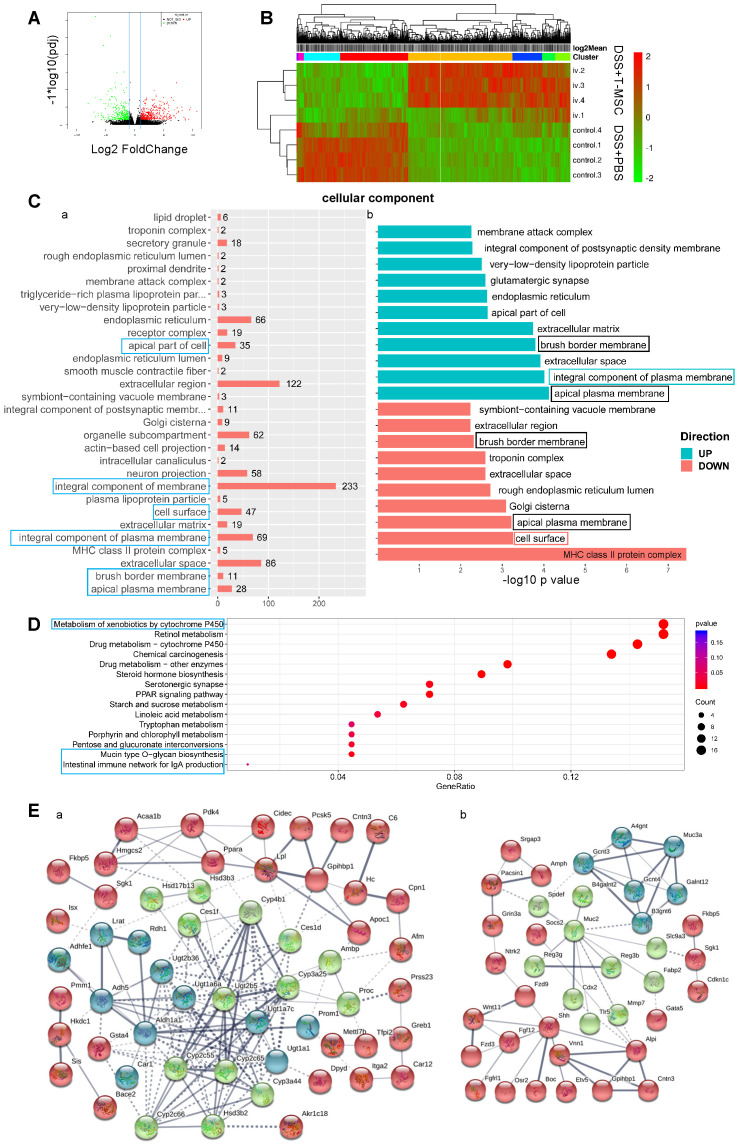
** RNA-sequencing of colon tissues.** Transcriptomic analysis of colon tissues in the acute colitis model was performed to investigate differential gene expressions at the transcriptional level. **A.** Volcano plot of DEGs. Red dots for up-regulated genes and green dots for down-regulated genes in the T-MSC group. **B.** Heatmap of hierarchical clustering of samples and DEGs. **C.** Gene Ontology enrichment analysis of DEGs in Cellular Component. a. GO annotations of DEGs and their corresponding gene numbers. Blue boxes represent annotations that might be related to T-MSC's therapeutic function. b. GO annotations of up-regulated (green) and down-regulated (red) DEGs. -log 10 *p*-value was used to measure the expressions of DEGs. The green box represents associated annotation with up-regulated DEGs, the red box represents associated annotation with down-regulated DEGs, and black boxes represent associated annotations with both up-regulated and down-regulated DEGs. **D.** KEGG pathway dot plot of DEGs. Dot size represents the numbers of DEGs, and the dot color represents the corresponding p value. Blue boxes represent pathways that might explain T-MSC's therapeutic function. **E.** PPI analysis of up-regulated DEGs. Line thickness indicates the strength of data support. a. proteins related to the detoxification process. b. proteins related to mucin function. DEG: differentially expressed gene; PPI: protein-protein interaction.

We performed RNA sequencing of colonic tissues in the acute DSS colitis model to elucidate the molecular basis of T-MSCs' therapeutic function. Sequenced reads were mapped to the mouse genome, and significantly differentially expressed genes (DEGs) were defined by the adjusted *p*-value (p adj) <0.05 and Fold-Change (FC)>2. A total of 677 DEGs were identified with 408 up-regulated and 269 down-regulated (**Figure 6A**). The hierarchical clustering of samples and DEGs showed differential expression between the treatment and control groups (**Figure 6B**). Gene Ontology (GO) analysis showed that DEGs were mostly enriched in annotations within the Cellular Component (CC), especially in integral components of the plasma membrane, including up-regulated genes in the T-MSC group (**Figure 6C**). Compared with the PBS control, up-regulated DEGs were associated with intestinal absorption, oligosaccharide binding, and toxic substance responses, while down-regulated DEGs were related to the MHC II protein complex and antigen presentation via MHC II. Annotations such as the apical plasma membrane and brush border membrane were found in both up-regulated and down-regulated genes (**Figure 6C, Figure S8A, B**)*,* indicating that the T-MSC injection might have contributed to colonic cell integrity, especially in maintaining an integral and homeostatic cell membrane. The Kyoto Encyclopedia of Genes and Genomes (KEGG) analysis showed that DEGs were enriched in pathways associated with metabolism, especially the metabolism of xenobiotics (**Figure 6D**). This could be explained by colonic cells maintaining normal functions by accelerating the metabolism of harmful components in the T-MSC group. Pathways related to cell apoptosis and mucin synthesis were also involved (**Figure S8C**). Protein-protein interaction (PPI) analysis indicated that up-regulated DEGs in the T-MSC group were related to the detoxification process and mucin production (**Figure 6E**), also contributing to the integrity of the mucosal barrier 30. Together, transcriptome analysis indicated that T-MSC treatment might maintain the integrity of colonic cells and promoted the metabolism of xenobiotics, which could be a prerequisite for the regeneration potential of intestinal epithelium.

### *In vitro* IGF-1 stimulation on colonic cell function and organoid growth

**Figure 7:**
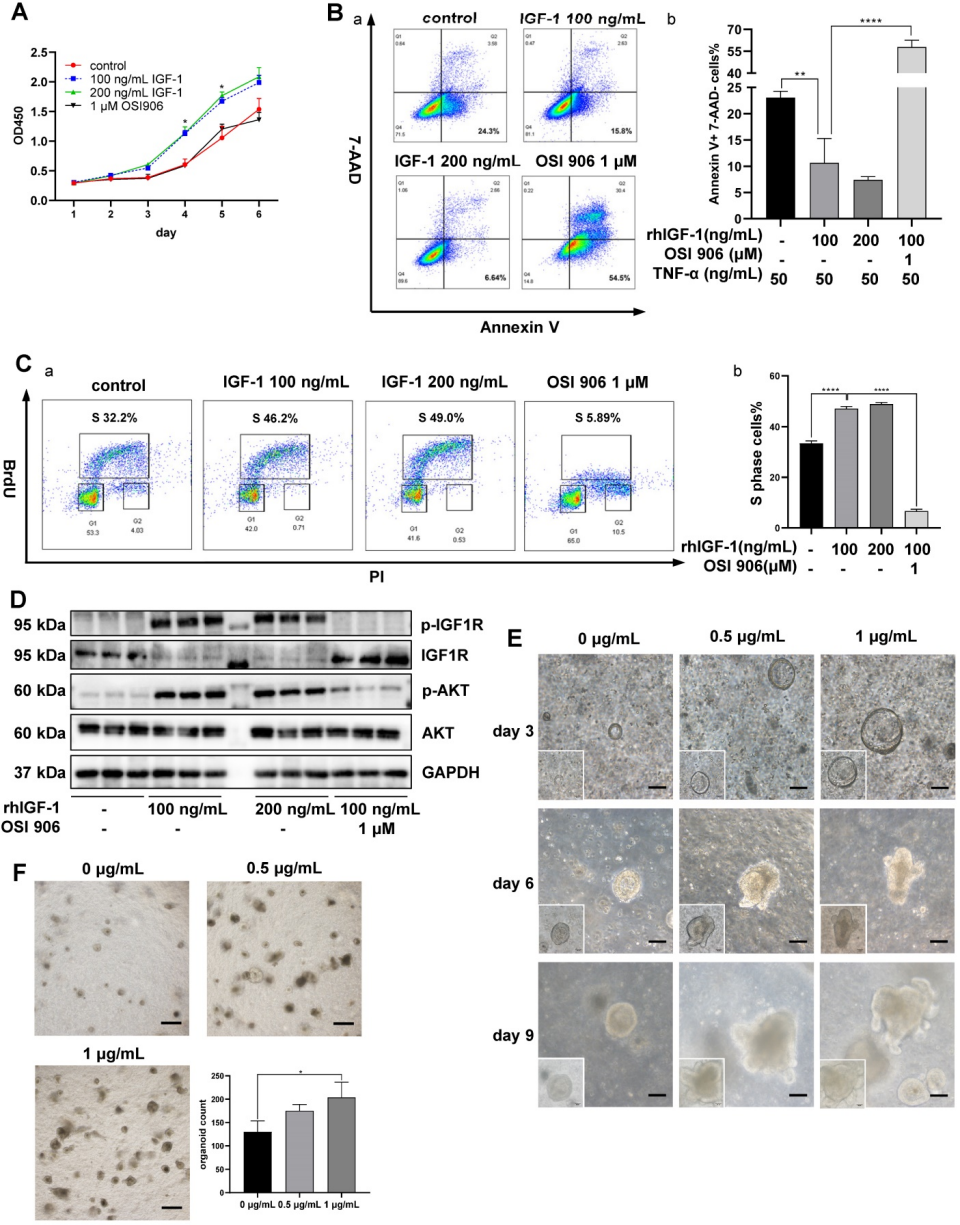
** Effects of *in vitro* IGF-1 stimulation on colon cells and organoids. A.** The proliferation of NCM 460 cells was measured by the CCK-8 assay. OD450 values were observed for 6 days. rhIGF-1 stimulation (100 ng/mL, 200 ng/mL) increased OD 450 values on day 4 and day 5. **B.** Apoptosis of NCM 460 cells was induced by 50 ng/mL TNF-α in culture media. Flow cytometry analysis of Annexin V and 7-AAD staining showed rhIGF-1 stimulation (100 ng/mL, 200 ng/mL) decreased the percentage of Annexin V+, 7-AAD- early apoptotic cells. **C.** Flow cytometry analysis of BrdU and PI staining was used to measure the cell cycle. rhIGF-1 stimulation (100 ng/mL, 200 ng/mL) increased BrdU+ S phase cell percentage compared with controls. **D.** Protein expressions of downstream IGF1-IGF1R pathway in NCM 460 cells were measured by Western blotting. rhIGF-1 stimulation increased the phosphorylation of AKT and IGF-1 receptors. **E.** Mice colon crypts were isolated for organoid culture and rmIGF-1 (0.5 µg/mL, 1 µg/mL) was added daily from day 3 (the time point when colon organoids began to appear and grow). *In vitro* IGF-1 stimulation increased the growth speed and led to a larger size and more buddings of the organoids. Scale bar = 100 µm **F.** Colon organoids were harvested and counted on day 10. rmIGF-1 stimulation increased total organoid numbers. Scale bar = 500 µm. Data are expressed as mean ± SD. *p < 0.05, **p < 0.01 and ****p < 0.0001. rhIGF-1: recombinant human IGF-1; rmIGF-1: recombinant mouse IGF-1.

Animal experiments indicated that IGF-1 might be the key factor promoting the regeneration and repair of colonic mucosa in DSS-induced colitis. We used human colon epithelial cell line NCM460 for *in vitro* IGF-1 stimulation to monitor cell functions. CCK-8 assay indicated that IGF-1 promoted proliferation of NCM460 cells, as observed by higher OD450 values than the control group (**Figure 7A**). To determine the influence of IGF-1 stimulation on cell death, cell apoptosis was induced by adding 50 ng/mL TNF-α to the culture medium. Flow cytometry analysis showed a decreased percentage of Annexin V (+) 7-AAD (-) apoptotic cells (**Figure 7B**) and an increased proportion of Annexin V (-) 7-AAD (-) live cells in the IGF-1 group. (**Figure S9A**). The cell cycle was measured by *in vitro* BrdU incorporation. Flow cytometry analysis found that exogenous IGF-1 stimulation led to a higher proportion of S phase cells and a lower proportion of G1 phase cells (**Figure 7C, S9B**). OSI 906 abrogated IGF-1 functions and led to a high percentage of cell death and cell cycle arrest. These results suggested that *in vitro* IGF-1 stimulation promoted the proliferation of NCM 460 cells. Increased phosphorylation of IGF1R and AKT was also observed by immunoblotting (**Figure 7D, S9C**). Another human colon epithelial cell line FHC was used to verify IGF-1 functions on cell cycle and proliferation (**Figure S10**).

To mimic the *in vivo* environment and investigate the effect of IGF-1 stimulation on intestinal epithelium directly, mouse colonic crypts were isolated and cultured at 500 crypts/well in a mixture of Matrigel and culture media. Colon organoids began to appear on day 3 at which time recombinant mouse IGF-1 was added daily. IGF-1-treated organoids were larger in size and showed more buddings than the control group (**Figure 7E**). When organoids in each well were counted on day 10, we found that IGF-1 stimulation increased the number of organoids (**Figure 7F**). However, PCNA staining showed no difference between IGF-1 and control groups, where all organoids possessed high proliferative potencies. (**Figure S9D**).

**Figure 8:**
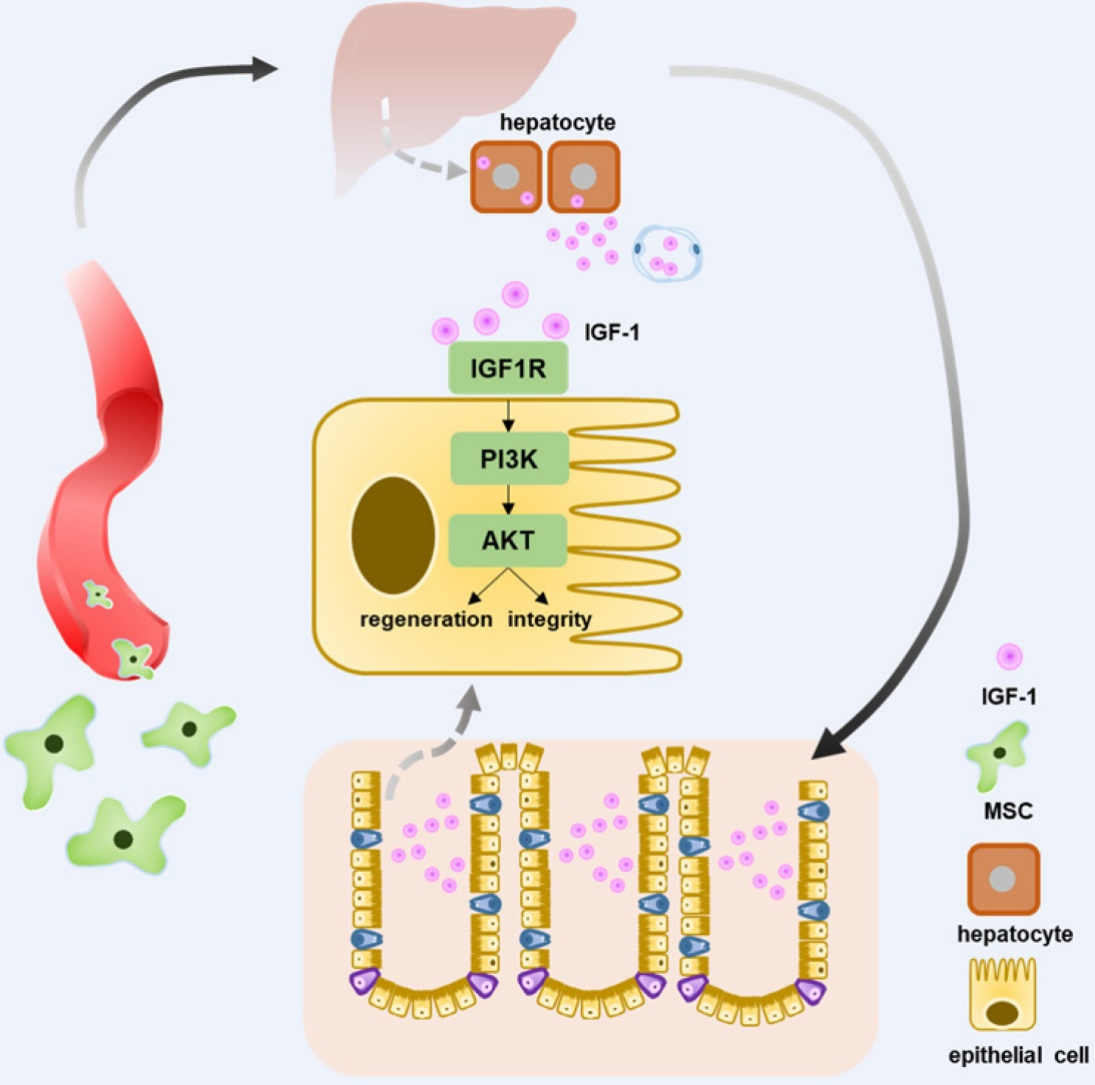
** Schematic diagram elucidating T-MSCs' therapeutic efficacy in colitis mice.** Intravenous injection of T-MSCs showed therapeutic function in both acute and chronic DSS-induced colitis. Antibody array test indicated T-MSCs injection increased serum IGF-1 level. Further study showed elevated serum IGF-1 might originated from endogenous secretion of the liver. IGF1R-PI3K-AKT pathway in colon epithelium was upregulated after T-MSCs treatment. RNA sequencing analysis of colon samples identified DEGs that were related to cell integrity, xenobiotic metabolism, and mucous barrier. In conclusion, increased IGF-1 contributed to the integrity and regeneration of colon epithelium and promoted the recovery of chemically induced colitis in mice.

In conclusion, intravenous injection of T-MSCs showed therapeutic potential in DSS colitis. Injected T-MSCs were mainly distributed in the lungs, liver, and spleen. Furthermore, immunofluorescence staining indicated that the cells were trapped in the lung vessels or showed co-localization with macrophages. Antibody microarray analysis showed that T-MSC treatment increased serum IGF-1 level, possibly originating from the liver. Application of IGF-1 receptor inhibitors blocked the therapeutic efficacy of T-MSCs, while exogenous IGF-1 administration reduced the inflammation. Elevated IGF-1 helped to maintain the integrity of colonic epithelial cells promoting its repair and regeneration via the IGF1R-PI3K-AKT pathway (**Figure 8**).

## Discussion

MSC populations possess heterogeneous biological characteristics that may influence their therapeutic potential in various disease models 31. MSCs from diverse tissue origins may present specific features, even within the same tissue type, donor condition, isolation method, *in vitro* culture and expansion procedures may also influence cell quality 32. Embryonic stem cell-derived MSCs are characterized by their uniform phenotypes, stable immunomodulatory properties, and trophic functions, and are amenable to large-scale culture. Their therapeutic potential in a multiple sclerosis (MS) animal model was reported 33. These properties make ESC-derived MSCs a promising candidate for off-the-shelf cell products in future clinical applications.

The *in vivo* distribution of T-MSCs after the intravenous injection appears to be advantageous for its potential therapeutic mechanisms. Accumulating evidence indicated that MSCs' therapeutic efficacy was not achieved by engraftment to the injured site but by their paracrine ability and cell surface markers, especially when they were injected systemically. Also, exosomes from MSCs enriched in proteins and microRNAs showed beneficial effects in animal disease models 34. In some cases, their immunomodulatory function was triggered via phagocytosis by mononuclear cells 35, consistent with the co-location of Dil-MSCs and macrophages in the liver observed in our study. We found that intravenously injected T-MSCs failed to migrate to the colon, while systemically infused T-MSCs increased IGF-1 levels in serum and colon and alleviated intestinal inflammation.

Intraperitoneal injection of MSCs has been reported to alleviate mouse experimental colitis by producing immunoregulatory molecules 36. We found that although intraperitoneal injection of T-MSCs failed to recover the body weight loss, it significantly decreased serum inflammatory cytokines. Therefore, the therapeutic function of intraperitoneal injection of T-MSCs still needs further exploration. Furthermore, it is also possible that this injection method will be effective if we increase the cell dose or injection numbers.

MSC-derived IGF-1 plays a therapeutic role in various disease models, as reported in many studies. MSC conditioned media enriched in IGF-1 and other immunomodulatory molecules contributed to regulating cell cycle, intestinal stem cells and the pro/anti-inflammatory cytokine balance, thus alleviating radiation-induced intestinal injury 37. IGF-1-overexpressing MSCs accelerated the mobilization of bone marrow stem cells and promoted myocardial repair 38. MSC-derived IGF-1 also induced proximal tubular cell proliferation and protected mice from renal injury 26. However, IGF-1's function in IBD is rarely discussed. Clinical studies confirmed that the serum IGF-1 was reduced in IBD patients, and prednisolone treatment increased IGF-1 level 39. IGF-1 also demonstrated potent trophic functions in parenterally fed rats by promoting epithelial cell proliferation and reducing apoptosis 40. Besides, high-throughput sequencing revealed that a combination of IGF-1 and fibroblast growth factor 2 (FGF-2) enhanced the clonogenic capacity and cell diversity in mouse small intestine organoids 41.

IGF-1 promoted proliferation and reduced apoptosis of NCM 460 and FHC cells *in vitro*. Exogenous IGF-1 stimulation also increased the number and size of mice colon organoids. We speculated that IGF-1 originated in the liver and confirmed this stance by real-time PCR, IHC staining, and ELISA, showing increased IGF-1 expression at transcriptional and translational levels. The co-culture of T-MSCs and hepatocytes also indicated that T-MSCs increased the secretion of mouse IGF-1. These results suggested that intravenously administrated T-MSCs stimulated endogenous IGF-1 expression in mice. Nevertheless, the specific mechanisms of how T-MSCs promoted host IGF-1 secretion remained unknown. This could be due to the interaction between T-MSCs and hepatocytes since a small fraction of T-MSCs migrated to the liver, increasing the IGF-1 secretion level. It was also possible that T-MSCs secreted specific proteins since co-culture in the Transwell system also increased the IGF-1 level. Genetically engineered MSCs with overexpression of insulin-like growth factors have been used in some disease models to improve MSC functions 42. With this finding that T-MSCs without overexpression of IGF-1 can promote the IGF-1 expression by the host, IGF-1 engineering of MSCs is not required.

Besides increasing endogenous IGF-1 level, whether other mechanisms were involved in T-MSC's therapeutic function still need further investigation. Although intravenous injection of 4 mg/kg IGF-1 alleviated colon inflammation, it was less effective than intravenous T-MSC injection. Thus, T-MSC may possess multiple beneficial functions in the treatment of experimental colitis. Further explorations are needed to clarify these mechanisms, especially the immunomodulatory properties.

Colon epithelial regenerative ability plays a key role in protecting from damages and inflammation caused by microbial flora or other injurious agents 43. Epithelial regeneration involves a complex process with various cell types and signaling pathways. A small population of undifferentiated and mitotically active crypt base columnar cells, possess stem cell identity and can proliferate and differentiate into mature epithelial cells to maintain the epithelium integrity 44. MSC therapy increased the non-canonical WNT4 ligand expression by epithelial cells and induced stimulation of endogenous host progenitor cells 45. We also discovered that T-MSC treatment maintained the integrity and proliferation potential of colonic epithelial cells. IGF-1 was increased, and its downstream IGF1R-PI3K-AKT pathway was up-regulated in the colonic epithelium. RNA sequencing revealed that DEGs were enriched in the integral component of the membrane, and PPI analysis indicated that the mucous barrier and detoxification process might be related to T-MSC's therapeutic function. The changes identified by the sequencing analysis need to be further verified.

Colonic epithelium repair is also closely related to its local stem cells. Two types of intestinal stem cells are associated with the epithelial renewal process. Lgr5+ stem cells located at the base of the crypt are classic intestinal stem cells that target the Wnt-β-catenin pathway and play an essential role in the regeneration of colonic mucosa 46. Recently, slow-cycling +4 stem cells featured by Hopx1, Lrig1, TERT, and Bmi1 illustrated their functions in promoting intestinal regeneration 47. In our study, increased IGF-1 promoted epithelium regeneration. IHC staining of Ki-67, BrdU, and Bmi1 indicated that colonic epithelium possessed better proliferative potential after T-MSC treatment. However, whether intestinal stem cells and Wnt signaling pathways are involved in the regeneration phase remains unclear and will be investigated in the future.

## Conclusions

Our study demonstrated that intravenous injection of T-MSCs alleviated both acute and chronic DSS-induced colitis in mice. *In vivo* tracking experiment indicated that injected cells were located in the lungs, liver, and spleen. Serum antibody array analysis and subsequent ELISA confirmed that IGF-1 was increased in T-MSC-treated mice, and administration of IGF-1 receptor inhibitors blocked T-MSCs' therapeutic functions. The remaining colon epithelium in the treatment group showed better proliferative potential and up-regulated IGF1R-PI3K-AKT expression. RNA sequencing analysis identified DEGs that were related to cell integrity, xenobiotic metabolism, and mucous barrier. *In vitro* IGF-1 stimulation also promoted cell proliferation and reduced apoptosis. Our study found that ESC-derived MSCs significantly increased endogenous IGF-1 secretion and maintained the integrity and regeneration potential of colonic epithelium, a strategy that could be used in future clinical applications.

## Supplementary Material

Supplementary figures and tables.

## Abbreviations

ANOVA: analysis of variance
BCA: bicinchoninic acid
CD: Crohn's disease
DAB: diaminobenzidine
DAI: disease activity index
DEG: differentially expressed gene
DSS: dextran sulfate sodium
ESC: embryonic stem cell
ELISA: enzyme-linked immunosorbent assay
FGF-2: fibroblast growth factor 2
FITC: fluorescein isothiocyanate
GO: gene ontology
HAI: histopathology activity index
HBSS: Hanks balance salt solution
HE staining: hematoxylin eosin stain
IBD: inflammatory bowel disease
IDO: indoleamine 2:3-Dioxygenase
IGF-1: insulin-like growth factor-1
IGFBP: insulin-like growth factor binding protein
ip: intraperitoneal
iv: intravenous
IHC: immunohistochemistry
KEGG: Kyoto encyclopedia of genes and genomes
MAPK: mitogen activated protein kinase
MSC: mesenchymal stem cell
mTOR: mammalian target of rapamycin
NC: negative control
NO: nitric oxide
PGE2: prostaglandin E2
PPP: picropodophyllin
PPI: protein-protein interaction
PI3K: phosphatidylinositol 3-kinase
RCT: randomized controlled trial
RT-PCR: real-time polymerase chain reaction
TSG-6: TNF-Stimulated Gene 6
UC: ulcerative colitis
