# Supplementary material for: Embryonic stem cell-derived mesenchymal stem cells promote colon epithelial integrity and regeneration by elevating circulating IGF-1 in colitis mice
Source: Theranostics. 2020 Oct 30;10(26):12204–22. doi: 10.7150/thno.47683 (PMC7667691; doi:10.7150/thno.47683)
Supplement: Supplementary file 1 — Supplementary figures and tables. [file thnov10p12204s1.pdf]

Supplementary Material

Embryonic stem cell-derived mesenchymal stem cells promote colon epithelial integrity and regeneration by elevating circulating IGF-1 in colitis mice

Jun Xu, Xiaofang Wang, Jiaye Chen, Shengbo Chen, Zhijun Li, Hongbin Liu, Yang Bai, Fachao Zhi.

Supplementary methods:

Animal experiments

Supplementary Tables:

Table S1: Scoring systems for DAI and HAI

Table S2: Primers used in reverse transcriptase polymerase chain reaction

Table S3: Serum microarray cytokines

Supplementary Figures:

Figure S1-S10

Supplementary Methods

Animal experiments

To compare the therapeutic efficacy of different administration approaches, acute DSS colitis was induced as described in Methods. Mice received an intravenous injection of 5×10^5 T-MSCs or intraperitoneal injection of 1×10^6 T-MSCs on day 3 and the body weight was recorded daily. Mice were sacrificed on day 9, and serum cytokines were measured using Mouse Th1/Th2/Th17 Cytokine Kit (BD) according to the manufacturer's instructions.

To explore whether *in vitro* stimulation of inflammatory cytokines could influence the therapeutic efficacy of T-MSCs, cells were pretreated with 20 ng/mL IFN-γ or 50 ng/mL TNF-α.

T-MSCs were derived from human embryonic stem cells ESI053. Besides ESI053, another human embryonic stem cell line H9 was used to obtain MSCs similar to T-MSCs. The therapeutic efficacy of ESI053- and H9-derived MSCs were compared in the mouse acute colitis model by giving 5×10^5 MSCs on day 3, and the body weight was measured daily.

To further determine the origin of elevated IGF-1, T-MSCs were transfected with siRNA (Umine-bio) to down-regulate the expression of Igf-1 by lipofectamine 3000 (Thermo Fisher Scientific). The therapeutic efficacy of si-Igf1, si-NC, and T-MSCs was evaluated in the acute DSS colitis model.

In the IGF-1 treatment experiment, acute DSS colitis was induced as described above. Mice were treated with intravenous IGF-1 (2 mg/kg or 4 mg/kg) injection or intraperitoneal IGF-1 (2 mg/kg) injection and the therapeutic efficacy was compared with the T-MSC group.

Supplementary Tables

Table S1 Scoring systems for DAI and HAI

a. Scoring system for DAI

Score	Weight loss	Stool consistency	Blood
0	None	Normal	Negative hemocult
1	1-5%	Soft but still formed	Negative hemocult
2	6-10%	Soft	Positive hemocult
3	11-18%	Very soft; wet	Blood traces in stool visible
4	>18%	Watery diarrhea	Gross rectal bleeding

b. Scoring system for HAI

Score	Tissue damage in DSS colitis	Lamina propria inflammatory cell infiltration in DSS colitis
0	None	Infrequent
1	Isolated focal epithelial damage	Increased, some neutrophils
2	Mucosal erosions and ulcerations	Submucosal presence of inflammatory cell clusters
3	Extensive damage deep into the bowel wall	Transmural cell infiltrations

Table S2: Primers used in reverse transcriptase polymerase chain reaction

Gene target	Forward primer	Reverse primer
GAPDH	AGGTCGGTGTGAACGGATTTG	TGTAGACCATGTAGTTGAGGTCA
IGF-1	ATAGAGCCTGCGCAATGGAA	GGCAGGGATAATGAGGCGAA
IGFBP1	ATCAGCCCATCCTGTGGAAC	TGCAGCTAATCTCTCTAGCACTT
IGFBP3	CCAGGAAACATCAGTGAGTCC	GGATGGAACTTGGAATCGGTCA
IGFBP4	AGAAGCCCCTGCGTACATTG	TGTCCCCACGATCTTCATCTT

Table S3: Serum microarray cytokines

**Microarray cytokines**					
AR	Axl	CD27L	CD30	CD40	CXCL16
EGF	E-selectin	Fractalkine	GITR	HGF	IGFBP-2
IGFBP-3	IGFBP-5	IGFBP-6	IGF-1	IL-12p70	IL-17E
IL-17F	IL-1ra	IL-2 Ra	IL-20	IL-23	IL-28
I-TAC	MDC	MIP-2	MIP-3a	OPN	OPG
Prolactin	Pro-MMP-9	P-selectin	Resistin	SCF	SDF-1a
TPO	VCAM-1	VEGF	VEGF-D	bFGF	BLC
CD30L	Eotaxin	Eotaxin-2	Fas L	G-CSF	GM-CSF
ICAM-1	IFNg	IL-1a	IL-1b	IL-2	IL-3

IL-4	IL-5	IL-6	IL-7	IL-10	IL-12p40
IL-13	IL-15	IL-17	IL-21	KC	Leptin
LIX	MCP-1	MCP-5	MCSF	MIG	MIP-1a
MIP-1g	PF4	RANTES	TARC	TCA-3	TNF RI
TNF RII	TNFa	4-1BB	ACE	ALK-1	CT-1
CD27	CD40L	CTLA4	Decorin	Dkk-1	Dtk
Endoglin	Fcg RIIB	Flt-3L	Galectin-1	Galectin-3	Gas 1
Gas 6	GITR L	HAI-1	HGF R	IL-1 R4	IL-3 Rb
IL-9	JAM-A	Leptin R	L-Selectin	Lymphotactin	MadCAM-1
MFG-E8	MIP-3b	Neprilysin	Pentraxin 3	RAGE	TACI
TREM-1	TROY	TSLP	TWEAK R	VEGF R1	VEGF R3
B7-1	BAFF R	BTC	C5a	CCL6	CD48
CD6	Chemerin	Clusterin	Lungkine	Cystatin C	DAN
DLL4	EDAR	Endocan	Fetuin A	H60	IL-33
IL-7 Ra	Kremen-1	Limitin	Lipocalin-2	LOX-1	Marapsin
MBL-2	Meteorin	Nope	NOV	Osteoactivin	OX40 Ligand
Periostin	PIGF-2	Progranulin	Prostasin	Renin 1	Testican 3
TIM-1	TRAIL	Tryptase ε	6Ckine	Activin A	ADAMTS1
Adiponectin	ANG-3	ANGPTL3	Artemin	CCL28	CD36
Chordin	CRP	E-Cadherin	Epigen	Epiregulin	Fas
Galectin-7	gp130	Granzyme B	Gremlin	IFNg R1	IL-17B
IL-17B R	IL-22	MIP-1b	MMP-2	MMP-3	MMP-10
PDGF-AA	Persephin	sFRP-3	Shh-N	SLAM	TCK-1
TECK	TGFb1	TRANCE	TremL1	TWEAK	VEGF-B
VEGF R2					

Supplementary Figures

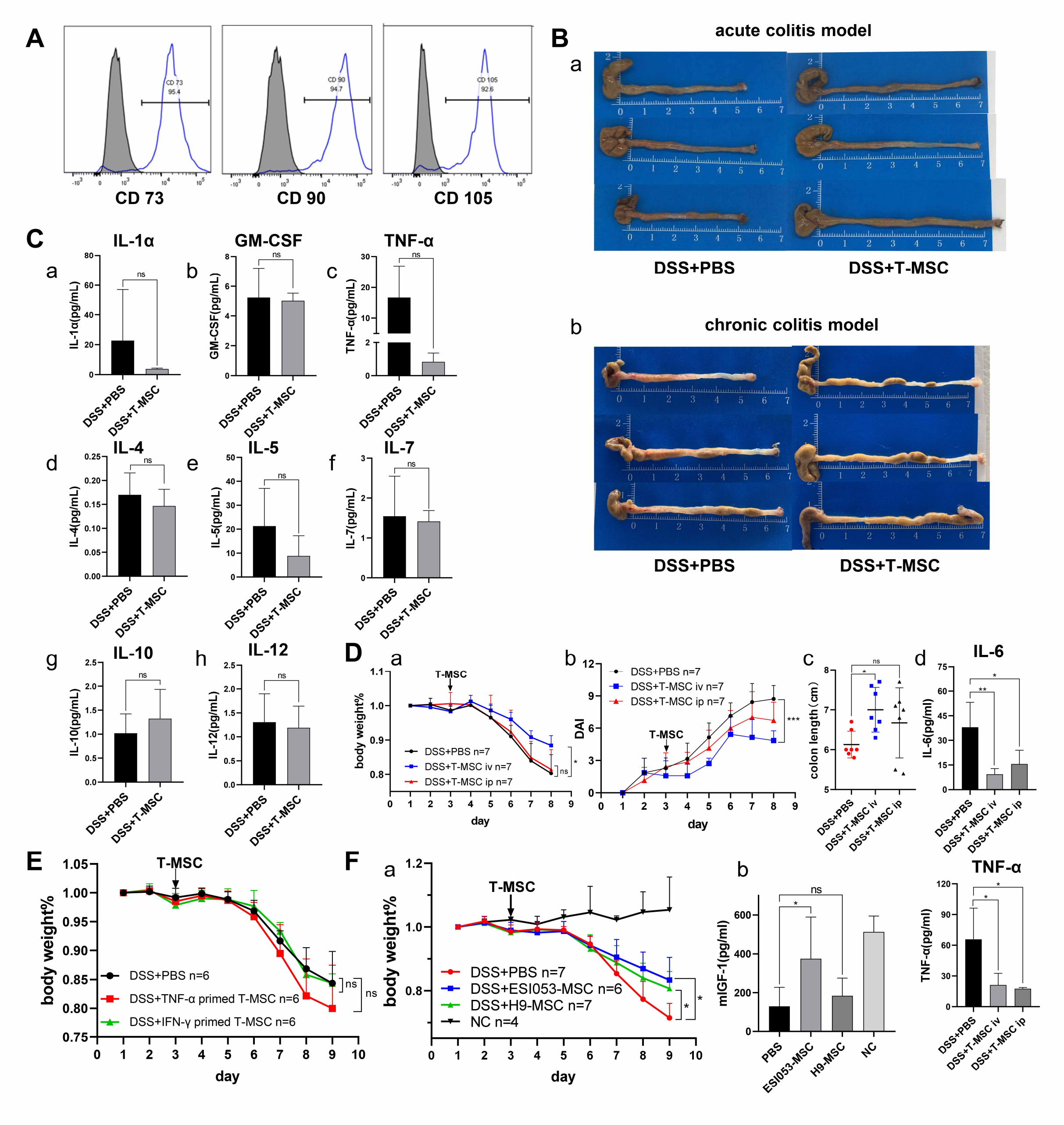

Figure S1

A. Phenotypic analysis of passage 5 T-MSCs by flow cytometry (gray line, negative staining control; blue line, surface markers). B. Photographs of mice colons from PBS and T-MSC groups. a. acute colitis model. b. chronic colitis model. C. Serum cytokines in acute DSS colitis model measured by the mouse high sensitivity T cell magnetic bead panel. D. Comparison of intravenous (iv) and intraperitoneal (ip) injection of T-MSCs in acute DSS colitis model. a. body weight percentage. b. DAI scores. c. colon length. d. inflammatory cytokines (IL-6 and TNF-α). E. Bodyweight percentage of DSS+PBS, DSS+TNF-α primed T-MSC, and DSS+IFN-γ-primed T-MSC groups. F. Comparison of

human embryonic stem cell line H9- and ESI053-derived T-MSCs for acute DSS colitis treatment. a. bodyweight percentage of DSS+PBS, DSS+ESI053 MSC, DSS+H9 MSC, and negative control groups. b. serum mouse IGF-1 was measured by ELISA. Data are expressed as mean ± SD. $^*p < 0.05$, $^{**}p < 0.01$ and $^{***}p < 0.001$.

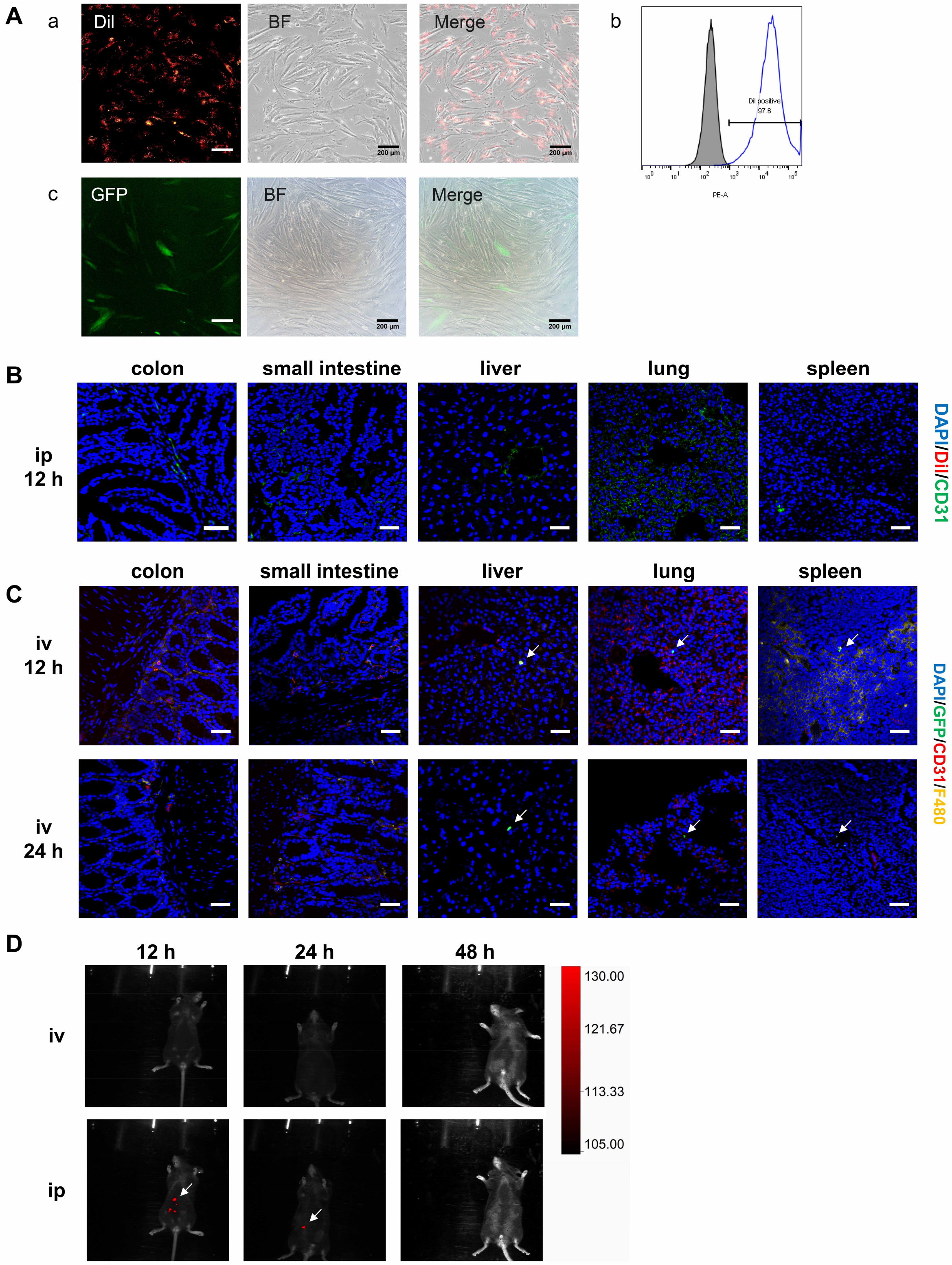

A
a
Dil
BF
Merge
200 μm
200 μm
b
Dil positive
97.6
PE-A
c
GFP
BF
Merge
200 μm
200 μm
B
colon
small intestine
liver
lung
spleen
ip
12 h
DAPI/DiI/CD31
C
colon
small intestine
liver
lung
spleen
iv
12 h
iv
24 h
DAPI/GFP/CD31/F480
D
12 h
24 h
48 h
iv
ip
130.00
121.67
113.33
105.00

Figure S2

A. a. fluorescent image (left), bright field image (middle), and merged image (right) of Dil labeled T-MSCs. b. flow cytometry analysis of Dil-labeled T-MSC (gray line, negative staining control; blue line, Dil staining). c. fluorescent image (left), bright filed image (middle) and merged image (right) of GFP labeled T-MSCs. Scale bar=200 μm B. Frozen sections of mice organs 12 h after intraperitoneal injection of Dil-labeled T-MSCs. Scale bar=50 μm C. *In vivo* distribution of GFP labeled T-MSCs 12 and 24 h after intravenous injection. Scale bar=50 μm. Arrows indicate labeled cell components. D. Bioluminescence imaging of luciferase-expressing T-MSCs. Arrows indicate luciferase-expressing T-MSCs.

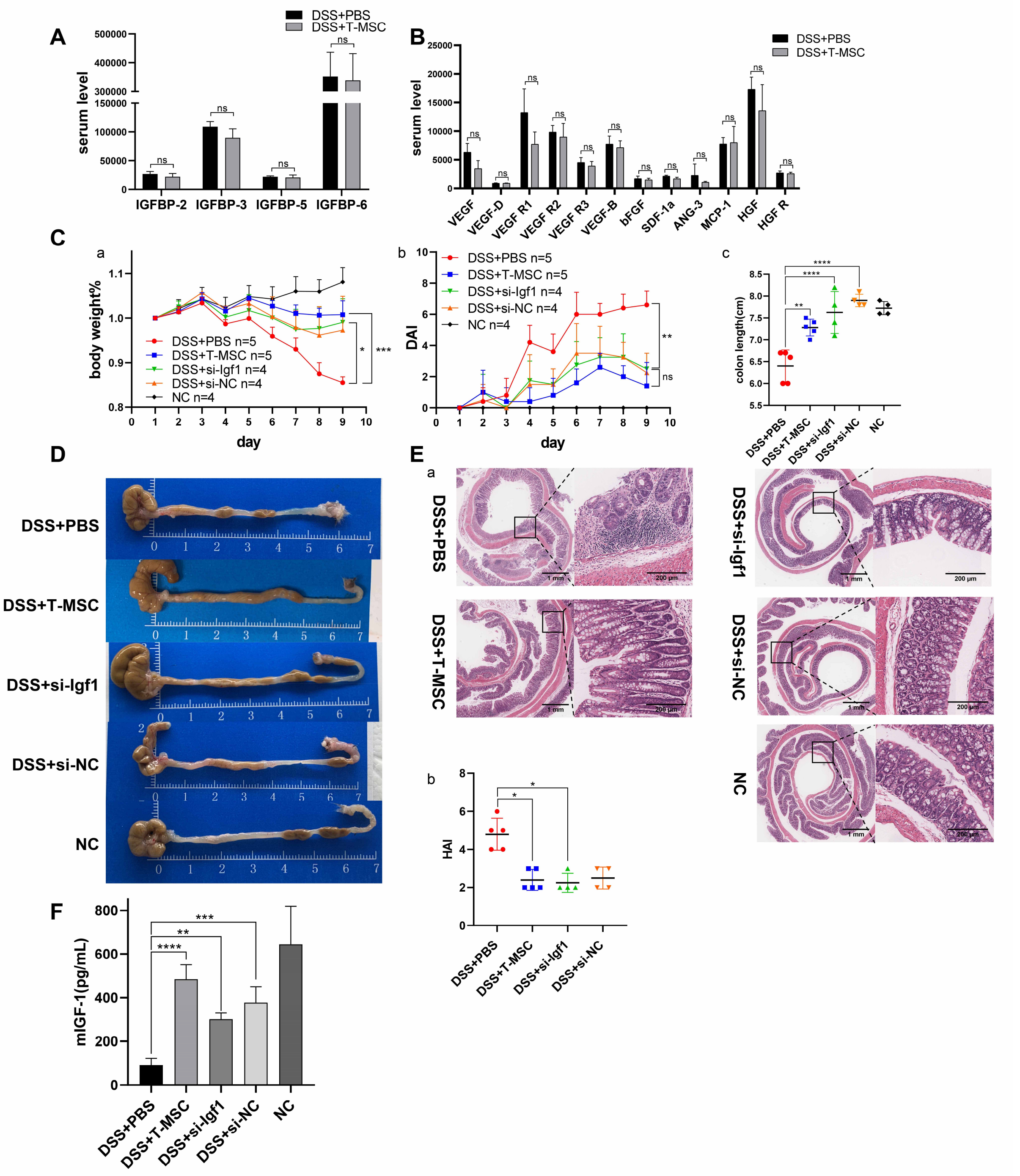

Figure S3

A. Antibody array tests of IGFBPs in mice serum samples. B. Antibody array tests of angiogenesis-related cytokines in mice serum samples. C. Therapeutic efficacy evaluation of si-Igf1 T-MSC. a. bodyweight percentage. b. DAI scores. c. colon length. D. Photographs of mice colons in each group. E. a. HE staining of colon sections in each group. b. HAI scores. Scale bar=1 mm left panel/200 μm right panel. F. Serum IGF-1 measured by ELISA. Data are expressed as mean ± SD. *p < 0.05, **p < 0.01, ***p < 0.001 and ****p<0.0001.

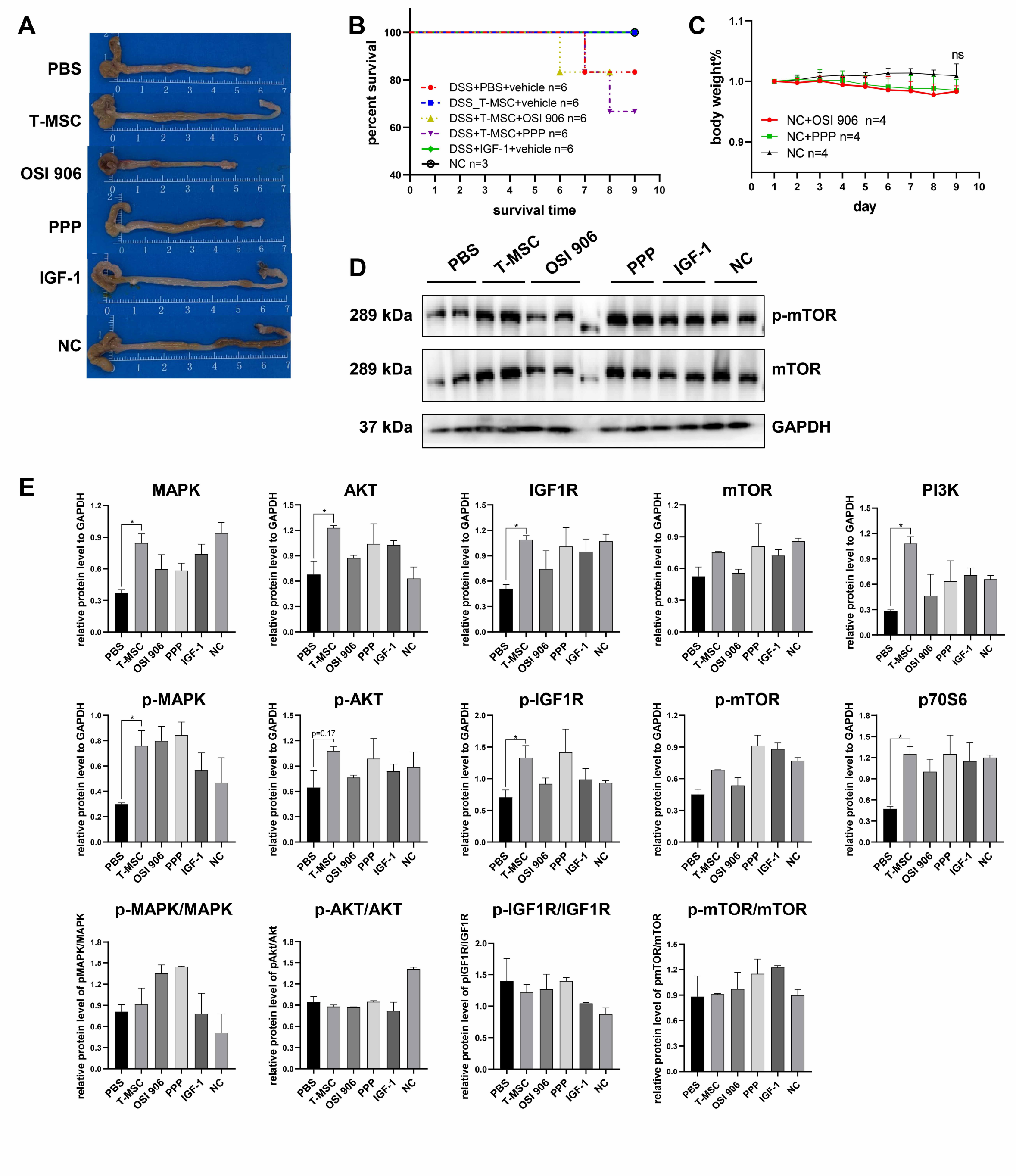

Figure S4

A. Photographs of mice colons in the IGF-1 receptor inhibitor model. B. Survival analysis of the IGF-1 receptor inhibitor model. C. Healthy untreated mice received a daily intraperitoneal injection of 30 mg/kg OSI 906 or PPP for 8 consecutive days. Bodyweight percentage was recorded and compared with untreated negative controls (n=4 in each group). D. Immunoblotting of mTOR and p-mTOR (downstream protein of PI3K-AKT pathway) in the IGF-1 receptor inhibitor group. E. Relative expression levels (normalized to GAPDH) of proteins from Figure 4E, S4D. Data are expressed as mean ± SD. *p < 0.05.

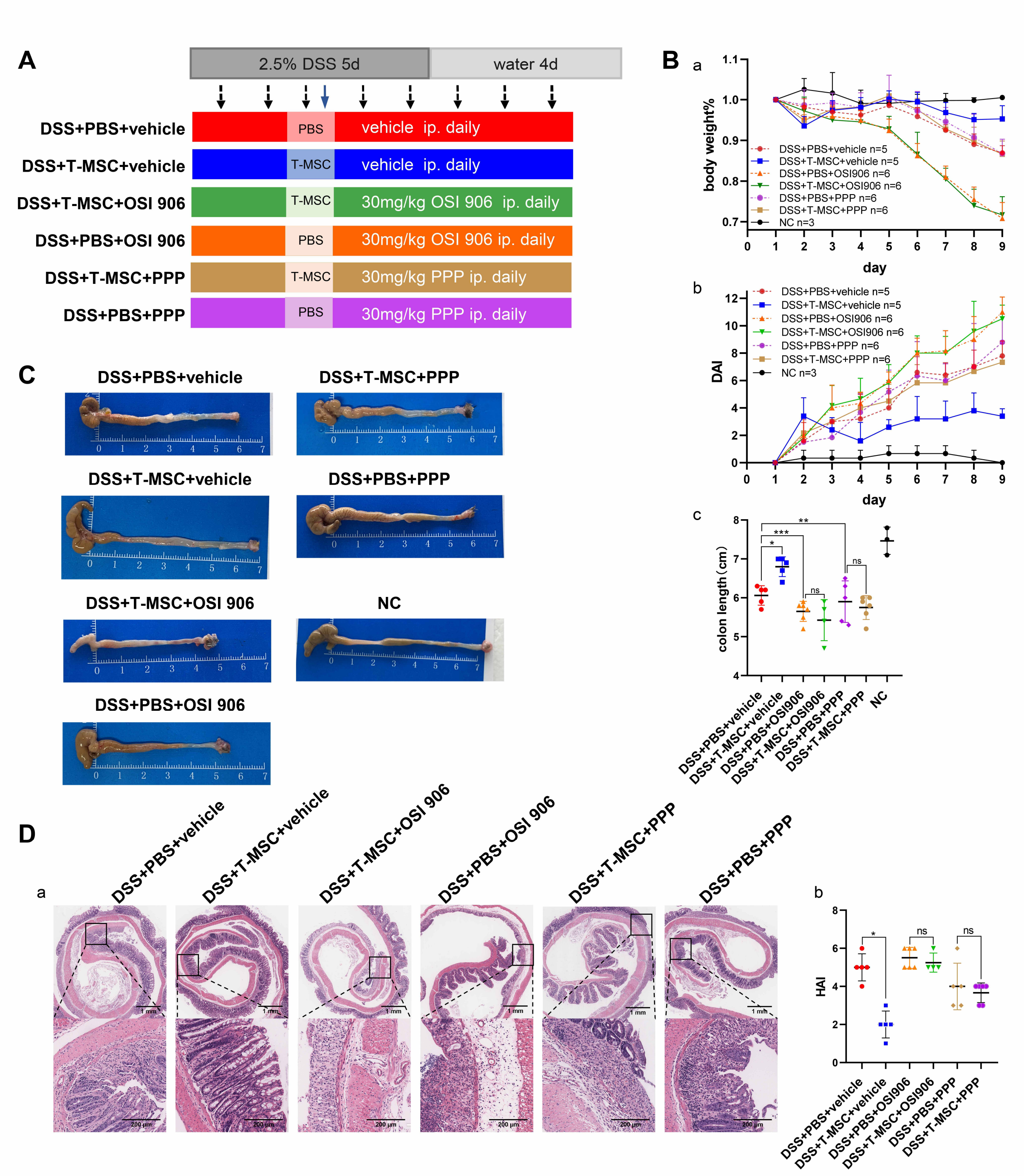

Figure S5

A. Schematic diagram of the experimental design. Acute colitis was induced by 2.5% DSS and mice were treated with DSS+PBS+vehicle, DSS+T-MSC+vehicle, DSS+T-MSC+inhibitor, and DSS+PBS+inhibitor, and therapeutic effects were measured and compared. B. Therapeutic efficacy was evaluated in each group. a. bodyweight percentage. b. DAI scores. c. colon length. C. Photographs of colons in each group. D. a. HE staining of colon sections in various groups. Scale bar=1 mm (top panel)/200 μm (bottom panel). b. HAI scores of colon sections. *p < 0.05, **p < 0.01 and ***p < 0.001.

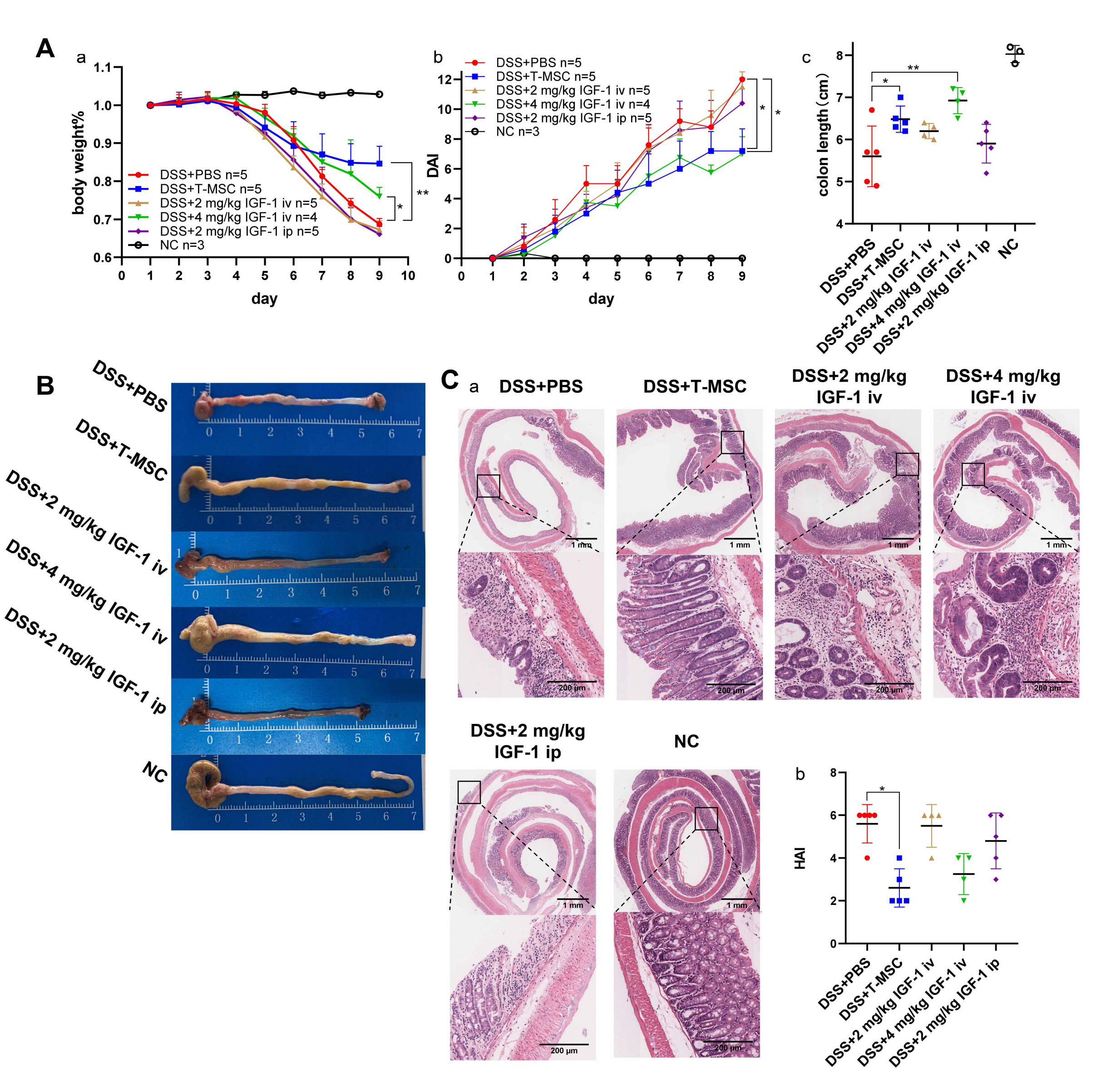

Figure S6

A. Acute colitis was induced as described in Figure S5, and mice were treated with 2 mg/kg or 4 mg/kg intravenous injection or 2 mg/kg intraperitoneal injection of mIGF-1, and the therapeutic efficacy was evaluated and compared with the T-MSC treatment group. a. bodyweight percentage. b. DAI scores. c. colon length. B. Photographs of mice colons in various groups. C. a. HE staining of colon sections in various groups. Scale bar=1 mm (top panel)/200 μm (bottom panel). b. HAI scores of colon sections. Data are expressed as mean ± SD. *$p < 0.05$ and **$p < 0.01$.

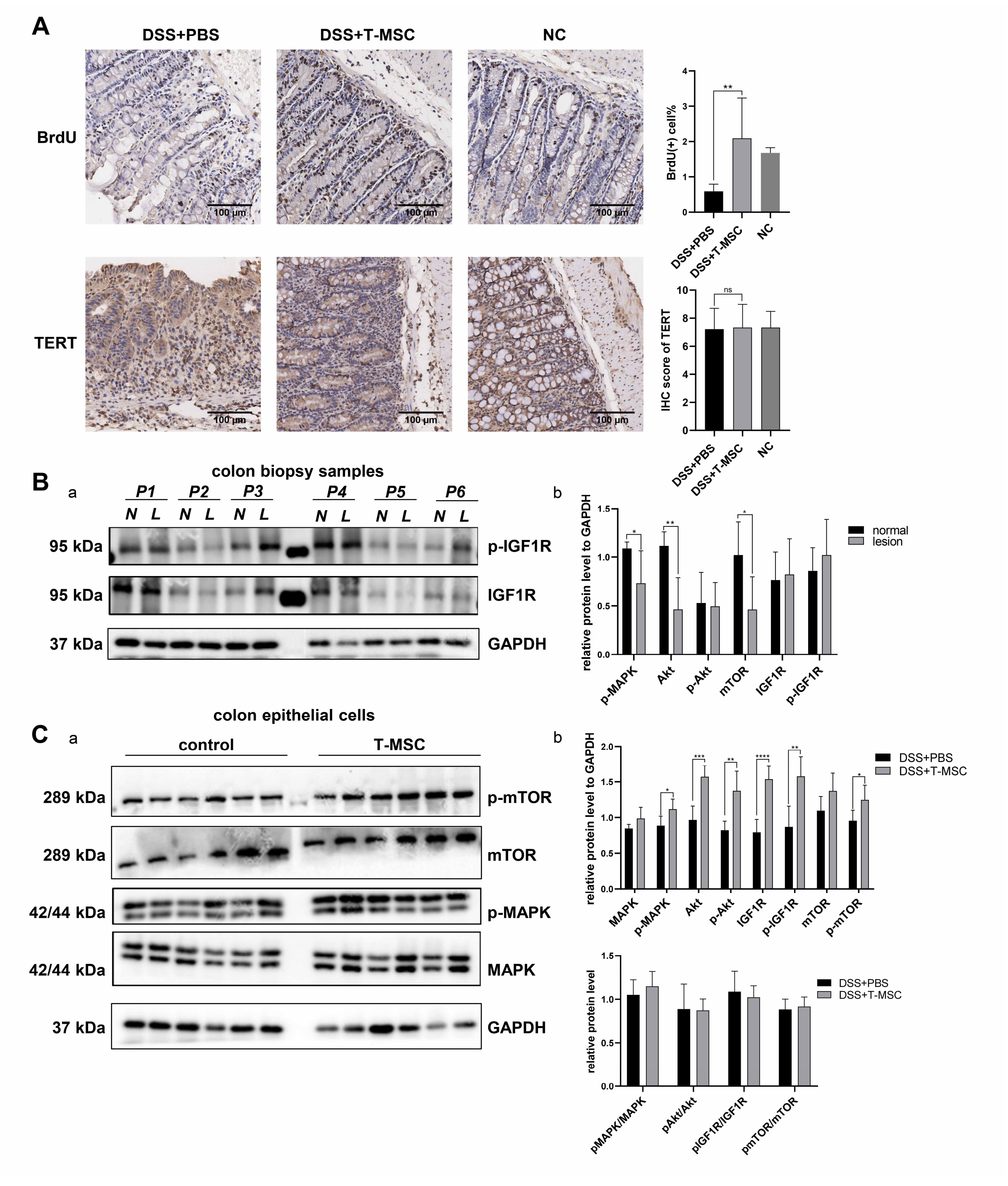

Figure S7

A. IHC staining of BrdU and intestinal stem cell marker TERT. Scale bar=100 μm. B. Western blotting of mucosa sample pairs of IBD patients. a. immunoblotting of IGF1R and p-IGF1R. b. relative protein expressions (normalized to GAPDH) of human colon mucosa samples in Figure 5C, S7B. C. Western blotting of mice colon epithelial cells. a. immunoblotting of MAPK, p-MAPK, mTOR, and p-mTOR. b. relative protein expressions (normalized to GAPDH) of mouse colon epithelial cells in Figure 5D, S7C. Data are expressed as mean ± SD. *$p < 0.05$, **$p < 0.01$, ***$p < 0.001$, ****$p<0.0001$.

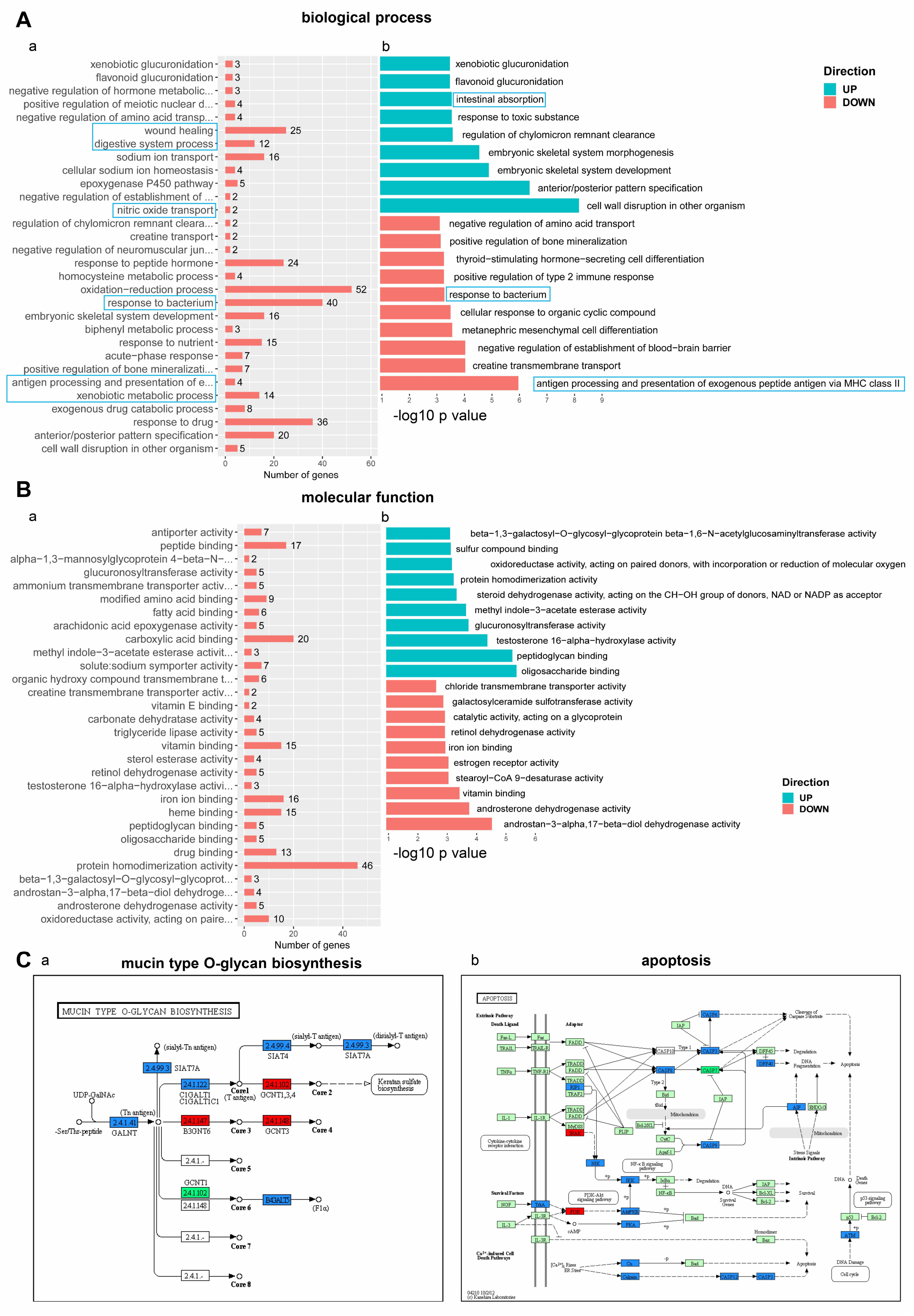
A
biological process
a
b
xenobiotic glucuronidation
flavonoid glucuronidation
negative regulation of hormone metabolic...
positive regulation of meiotic nuclear d...
negative regulation of amino acid transp...
wound healing
digestive system process
sodium ion transport
cellular sodium ion homeostasis
epoxygenase P450 pathway
negative regulation of establishment of ...
nitric oxide transport
regulation of chylomicron remnant cleara...
creatine transport
negative regulation of neuromuscular jun...
response to peptide hormone
homocysteine metabolic process
oxidation−reduction process
response to bacterium
embryonic skeletal system development
biphenyl metabolic process
response to nutrient
acute−phase response
positive regulation of bone mineralizati...
antigen processing and presentation of e...
xenobiotic metabolic process
exogenous drug catabolic process
response to drug
anterior/posterior pattern specification
cell wall disruption in other organism
Number of genes
intestinal absorption
response to toxic substance
regulation of chylomicron remnant clearance
embryonic skeletal system morphogenesis
embryonic skeletal system development
anterior/posterior pattern specification
cell wall disruption in other organism
negative regulation of amino acid transport
positive regulation of bone mineralization
thyroid−stimulating hormone−secreting cell differentiation
positive regulation of type 2 immune response
response to bacterium
cellular response to organic cyclic compound
metanephric mesenchymal cell differentiation
negative regulation of establishment of blood−brain barrier
creatine transmembrane transport
antigen processing and presentation of exogenous peptide antigen via MHC class II
-log10 p value
Direction
UP
DOWN
B
molecular function
a
b
antiporter activity
peptide binding
alpha−1,3−mannosylglycoprotein 4−beta−N−...
glucuronosyltransferase activity
ammonium transmembrane transporter activ...
modified amino acid binding
fatty acid binding
arachidonic acid epoxygenase activity
carboxylic acid binding
methyl indole−3−acetate esterase activit...
solute:sodium symporter activity
organic hydroxy compound transmembrane t...
creatine transmembrane transporter activ...
vitamin E binding
carbonate dehydratase activity
triglyceride lipase activity
vitamin binding
sterol esterase activity
retinol dehydrogenase activity
testosterone 16−alpha−hydroxylase activi...
iron ion binding
heme binding
peptidoglycan binding
oligosaccharide binding
drug binding
protein homodimerization activity
beta−1,3−galactosyl−O−glycosyl−glycoprot...
androstan−3−alpha,17−beta−diol dehydroge...
androsterone dehydrogenase activity
oxidoreductase activity, acting on paire...
Number of genes
beta−1,3−galactosyl−O−glycosyl−glycoprotein beta−1,6−N−acetylglucosaminyltransferase activity
sulfur compound binding
oxidoreductase activity, acting on paired donors, with incorporation or reduction of molecular oxygen
protein homodimerization activity
steroid dehydrogenase activity, acting on the CH−OH group of donors, NAD or NADP as acceptor
methyl indole−3−acetate esterase activity
glucuronosyltransferase activity
testosterone 16−alpha−hydroxylase activity
peptidoglycan binding
oligosaccharide binding
chloride transmembrane transporter activity
galactosylceramide sulfotransferase activity
catalytic activity, acting on a glycoprotein
retinol dehydrogenase activity
iron ion binding
estrogen receptor activity
stearoyl−CoA 9−desaturase activity
vitamin binding
androsterone dehydrogenase activity
androstan−3−alpha,17−beta−diol dehydrogenase activity
-log10 p value
Direction
UP
DOWN
C a
mucin type O-glycan biosynthesis
MUCIN TYPE O-GLYCAN BIOSYNTHESIS
b
apoptosis
APOPTOSIS

Figure S8

A. Gene Ontology enrichment analysis of DEGs in Biological Process annotation. a. GO annotations of DEGs and their corresponding gene numbers. b. GO annotations of up-regulated (green) and down-regulated (red) DEGs. B. Gene Ontology enrichment analysis of DEGs in Molecular Function annotation. a. GO annotations of DEGs and their corresponding gene numbers. b. GO annotations of up-regulated (green) and down-regulated (red) DEGs. -log 10 p-value was used to measure DEG expression. Blue boxes represent annotations that might be associated with T-MSC's therapeutic efficacy. C. KEGG pathways associated with DEGs. a. mucin-type O glycan biosynthesis. b. apoptosis. The red boxes represent up-regulated genes or proteins, the green boxes represent down-regulated genes or proteins, and the blue boxes represent mapped genes or proteins.

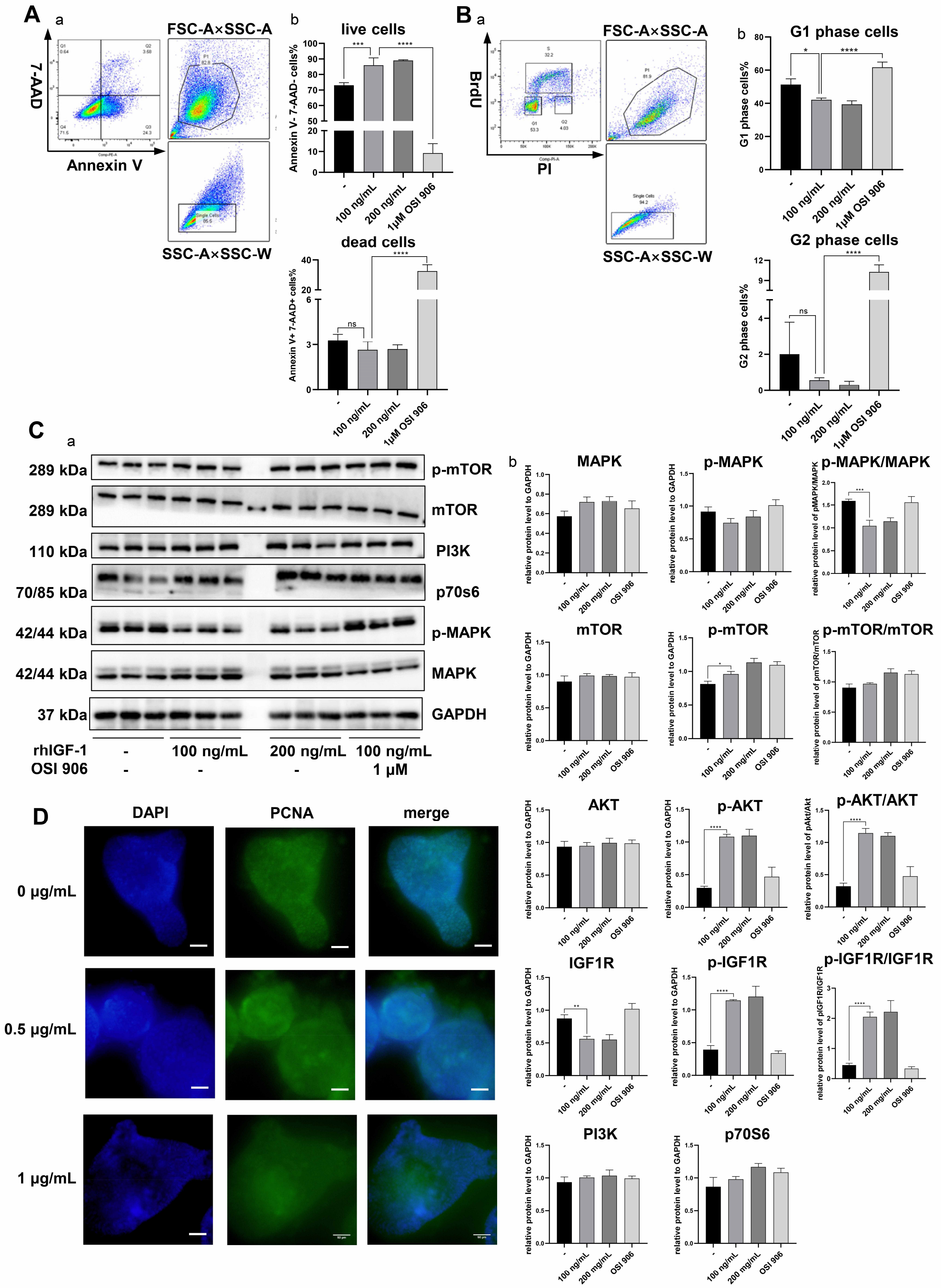

A
a
7-AAD
Annexin V
FSC-A×SSC-A
SSC-A×SSC-W
b
live cells
Annexin V- 7-AAD- cells%
dead cells
Annexin V+ 7-AAD+ cells%
ns
100 ng/mL
200 ng/mL
1μM OSI 906
B
BrdU
PI
G1 phase cells
G1 phase cells%
G2 phase cells
G2 phase cells%
C
289 kDa
p-mTOR
mTOR
110 kDa
PI3K
70/85 kDa
p70s6
42/44 kDa
p-MAPK
MAPK
37 kDa
GAPDH
rhIGF-1
OSI 906
100 ng/mL
200 ng/mL
1 μM
MAPK
p-MAPK
p-MAPK/MAPK
mTOR
p-mTOR
p-mTOR/mTOR
AKT
p-AKT
p-AKT/AKT
IGF1R
p-IGF1R
p-IGF1R/IGF1R
PI3K
p70S6
relative protein level to GAPDH
OSI 906
D
DAPI
PCNA
merge
0 μg/mL
0.5 μg/mL
1 μg/mL

Figure S9

A. Annexin V and 7-AAD staining of NCM 460 cells. a. gating strategy. b. live cell (Annexin V-, 7-AAD-) percentage and dead cell (Annexin V+, 7-AAD+) percentage in each group. B. BrdU staining of NCM 460 cells. a. gating strategy. b. G1 and G2 phase cell percentages in each group. C. Western blotting of NCM 460 cells. a. immunoblotting of MAPK, p-MAPK, mTOR, p-mTOR, p70s6, and PI3K. b. relative expression levels (normalized to GAPDH) of cell proteins in Figure 7D, S9C. D. PCNA staining of colon organoids. Scale bar=50 μm. Data are expressed as mean ± SD. *$p < 0.05$, **$p < 0.01$, ***$p < 0.001$, ****$p<0.0001$.

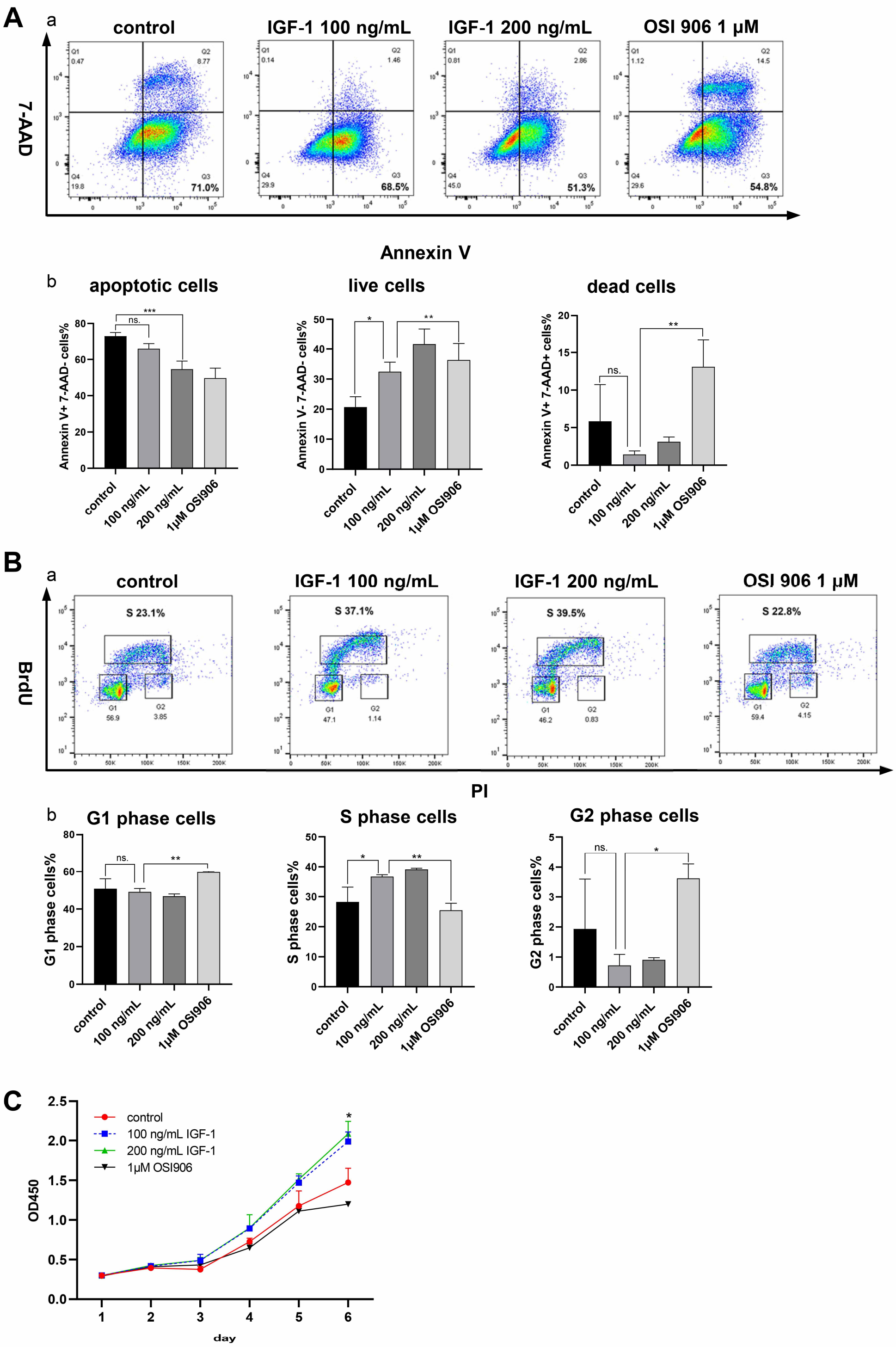

Figure S10

IGF-1 stimulation on cell cycle and proliferation in FHC cells. A. Cell apoptosis was induced by 50 ng/mL TNF-α and rhIGF-1 was added *in vitro*. a. flow cytometry analysis of Annexin V and 7-AAD staining. b. percentages of apoptotic cells, live cells, and dead cells in each group. B. The cell cycle was measured by BrdU incorporation. a. flow cytometry analysis of BrdU and PI staining. b.

percentages of G1, S, and G2 phase cells. C. Cell proliferation was measured by the CCK-8 assay. OD450 values were observed for 6 days; rhIGF-1 stimulation (100 ng/mL, 200 ng/mL) increased OD 450 values on day 6. Data are expressed as mean ± SD. *$p < 0.05$, **$p < 0.01$ and *** $p < 0.001$.
